# AllergoOncology: Role of immune cells and immune proteins

**DOI:** 10.1002/clt2.12133

**Published:** 2022-03-28

**Authors:** Mario Di Gioacchino, Loredana Della Valle, Alessandro Allegra, Giovanni Pioggia, Sebastiano Gangemi

**Affiliations:** ^1^ Center for Advanced Science and Technology G. d’Annunzio University Chieti Italy; ^2^ IDA – Institute of Clinical Immunotherapy and Advanced Biological Treatments Pescara Italy; ^3^ Division of Hematology Department of Human Pathology in Adulthood and Childhood ‘Gaetano Barresi’ University of Messina Messina Italy; ^4^ Institute for Biomedical Research and Innovation (IRIB) National Research Council of Italy (CNR) Messina Italy; ^5^ Department of Clinical and Experimental Medicine School of Allergy and Clinical Immunology, and Operative Unit of Allergy and Clinical Immunology University of Messina Messina Italy

**Keywords:** allergy, IgE, immune surveillance, immunomodulation, malignancy susceptibility

## Abstract

**Background:**

Immune cells and immune proteins play a pivotal role in host responses to pathogens, allergens and cancer. Understanding the crosstalk between allergic response and cancer, immune surveillance, immunomodulation, role of immunoglobulin E (IgE)‐mediated functions and help to develop novel therapeutic strategies. Allergy and oncology show two opposite scenarios: whereas immune tolerance is desired in allergy, it is detrimental in cancer.

**Aim:**

The current review provides an update on the role of immune cells and immune proteins in allergy and cancer fields.

**Methods:**

Authors investigated the role of relevant immunological markers and the correlation with cancer progression or cancer suppression.

**Results:**

Activated immune cells such as macrophages ‘M1’, dendritic cells (DCs), innate lymphoid cells (ILC2), NK cells, Th1, follicular T helper cells (TFH), TCD8+, B lymphocytes and eosinophils have inhibitory effects on tumourigenesis, while tolerogenic cells such as macrophages ‘M2,’ tolerogenic DCs, ILC3, T and B regulatory lymphocytes appear to favour carcinogenesis. Mastocytes and alarmins can have both effects. RIgE antibodies and CCCL5 chemokine have an anticancer role, whereas IgG4, free light chains, Il‐10, TGF‐β, lipocalin‐2, CCL1 chemokine promote cancer progression. Fundamental is also the contribution of epigenetic changes regulated by the microRNA in cancer progression.

**Conclusion:**

This knowledge represents the key to developing new anticancer therapies.

## 1 INTRODUCTION

Immune cells have a relevant role in the allergic process and take part in tumourigenesis. Stimulated immune cells, like classically activated macrophages ‘M1,’ activated dendritic cells (DCs), IL‐33 and amphiregulin, innate lymphoid cells (ILC2), NK cells, Th1, follicular T helper cells (T_FH_), IFN‐ϒ producing T CD8+ and B lymphocytes have inhibitory effects on tumourigenesis and tumour progression. While tolerogenic immune cells like alternatively activated macrophages ‘M2’ (M2a, M2b and M2c), tolerogenic DCs, ILC3, T regulatory and B regulatory lymphocytes, inhibiting allergic sensitization and response, appear to favour carcinogenesis. Furthermore, M2 subtypes macrophages (M2a, M2b), IL‐25 stimulated ILC2 and Th2 lymphocytes have a role both in inducing allergic reactions and in favouring cancer progression. Also, mast cells have a different effect on tumourigenesis based on multiple factors cancer‐related. Eosinophils have shown a prevalent tumouricidal function mediated by α‐defensins, TNF‐α, granzymes A and IL‐18.^1^ IgE antibodies showed anticancer role while IgG4 induce immune tolerance and represent an escape to antitumor immune response. Free light chains (FLCs), regulatory cytokines such as IL‐10, TGFβ, lipocalin‐2 (LCN‐2) and chemokines (e.g., CCCL1), promote cancer progression; however, CCCL5 chemokine has demonstrated an anticancer role. The group of alarmins (HGMB1, IL‐1α, S100 proteins and IL‐33) showed a different role, promoting or inhibiting tumour progression, depending on the type of the tumour, stage and their localization.^2^, ^3^ Finally, epigenetic changes regulated by microRNA (miRNA) exert a notable contribution to the immune response and cancer development.^4^

The European Academy of Allergy and Clinical Immunology established a Task Force on AllergoOncology to evaluate the relationships between cancer and allergy with the goal of studying both allergic problems in clinical oncology and the immunomodulatory mechanisms eventually protecting cancer to develop new oncological immunotherapy (e.g., cellular vaccines expressing IgE‐binding tumour antigens; recombinant antitumour IgE).^2^, ^3^ Studies in this field are constantly updated.

### 1.1 Epidemiologic association between allergy and cancer

Several epidemiological studies have suggested inverse associations between allergic diseases and malignancies. Allergy published, in 2005, two well‐documented studies on this topic. The first, carried out at the Stockholm Karolinska Institutet,^5^ analysed the possible presence of neoplasia and the allergic condition of 70,000 patients, reaching a neutral conclusion that allergy does not protect or promote the onset of tumours. The second, realized at the University of Heidelberg in Germany,^6^ was a review based on 80 previous epidemiological studies. In total, the clinical conditions of 52,000 patients were analysed, reaching a conclusion that allergy has a certain protective activity (actually, with some discrepancies) for tumours of colorectal cancer, breast, pancreas, brain (glioma, but not for meningioma) and leukaemia. On the contrary, allergy could be a risk factor for lung cancer. Other epidemiologic studies have explored the potential association between allergy history and cancer (first brain, lymphatic and haematopoietic cancers). However, most studies have relied on self‐reported allergic history, being typically limited, retrospective and associated with potential biases. Successive observations have reported an inverse association between allergy and colorectal carcinoma,^7^ but not with haematopoietic or prostate cancer.^8^, ^9^ One study reported an inverse trend between increasing blood eosinophil count and subsequent colorectal cancer risk.^10^

Other studies also evaluated biological indicators of allergy history and immune function. The level of total and specific IgE seems to have an inverse relationship with the development of neoplasia such as melanoma, glioma, gynaecological tumours and female breast cancer.^11^ A potential correlation between allergies and risk of haematologic malignancies (HMs) has been evaluated in numerous epidemiological analyses. The greater part of investigations has studied the relations between allergy and acute lymphoblastic leukaemia or lymphomas.^12^ A report suggests a relatively augmented risk of HMs in women but not in men with a story of allergies to airborne allergens, particularly to grass, plants or trees,^13^ while, in a population‐based report from the Swedish cancer registries, an augmented possibility of lymphoplasmacytic lymphoma was stated in subjects who had history of any form of allergy founded on previous hospital discharge information.^14^ Moreover, numerous findings indicate that asthma is a risk factor for acute leukaemia (AL) in children with Down syndrome, while skin allergies appeared to defend subjects from AL.^15^, ^16^ In a US veterans report, a history of total allergic situations as verified in the hospital records was correlated with a diagnosis of non‐Hodgkin's lymphoma (NHL). Important correlations were also reported in patients with allergic situations such as dermatitis, alveolitis and erythema, but not asthma.^17^ Different analyses suggest a positive correlation, especially for Hodgkin lymphoma (HL).^18^, ^19^ Finally, a possible correlation has been proposed between allergy and cutaneous lymphomas such as mycosis fungoides (MF). A story of allergic rhinitis was reported for 25.5% of patients with typical MF and 31% of subjects with atypical MF. However, the incidence of asthma and eczema was low. The total amount of IgE (IgE‐t) and eosinophil counts were greater for subjects with typical MF than for controls and for subjects with atopic diathesis than for subjects without atopy.^20^

Nevertheless, the findings were not always uniform. An inverse correlation was described primarily in case–control design analyses. For instance, an inverse relationship with a story of allergies has been stated for HM, lymphomas, HL, NHL, acute lymphoblastic leukaemia and multiple myeloma.^21^, ^22^, ^23^, ^24^, ^25^, ^26^, ^27^, ^28^, ^29^, ^30^ The correlation between allergy and AL proposes that the two pathologies may have a shared biologic mechanism. Two hypotheses, such as ‘missing immune deviation’ and ‘decreased immune suppression,’ have been suggested to elucidate the biological basis of this assumption. Epidemiological analyses have demonstrated that changes of a microbial exposure are the main element motivating the rising frequency of atopic diseases (the so‐called ‘hygiene hypothesis’).^31^ However, the immunological cause of this hypothesis is still debatable. The early explanations established that a deficiency of shifting of allergen‐specific reactions from the Th2 to the Th1 phenotype (missing immune deviation) was responsible. This signifies that decreased stimulation of Toll‐like receptors on natural killer (NK) cells and DCs induces a reduced generation of cytokines, such as IFN‐α and IFN‐γ, and IL‐12 which not only stimulate the expansion of Th1 cells but also disturbed the growth of Th2 cells. Lately, however, the relevance of diminished action of T cells (reduced immune suppression) has been underlined. Agreeing to this theory, the reduced microbial burden does not operate by causing a diminished generation of Th1‐polarizing cytokines, but by reducing the effects of Treg cells.^32^ In both cases, there is a profound alteration in the functionality of the immune system. Moreover, to clarify the role of allergies as a risk factor for haematological diseases, it is possible considering the antigenic stimulation theory which suggests that chronic stimulation of the immune system will cause accidentally occurring pro‐oncogenic mutations in proliferating cells.^33^ In contrast, allergies as protective factors can be justified in terms of the immune‐surveillance theory, which propose that allergic pathologies increase the immune system's capability to identify and eradicate neoplastic cells. However, more studies will be necessary to define which structure of the immune system of allergic patients may constitute a risk factor for haematological neoplasms.

At present, no conclusive results have been achieved and literature data are inconsistent and contradictory, suggesting the importance of immuno‐epidemiology studies on cancer, that consider other interfering factors such as environment, lifestyle, age, sex, job, alcohol, smoking use, type and duration of allergic disease other than the simple allergic status.

## 2 ROLE OF IMMUNE CELLS IN ALLERGY AND CANCER

### 2.1 Macrophages

Macrophages are an essential component of innate immunity and play a leading role in inflammation and host defence.^34^ They explicate their role through phagocytosis and generate reactive oxygen species (ROS), nitrogen intermediates and other cytotoxic factors. ROS and nitrogen intermediates are also responsible for the exacerbation of allergy and asthma severity.^35^ They are professional antigen‐presenting cells (APCs) involved in allergy and autoimmune response, especially in delayed‐type hypersensitivity. Due to various signals, macrophages may undergo classical M1 activation (stimulated by TLR ligands such as LPS and IFN‐ϒ) or alternative M2 activation (stimulated by IL‐4/IL‐13). The M1 phenotype expresses elevated levels of proinflammatory cytokines, reactive nitrogen and oxygen intermediates promote Th1 response and strong microbicidal and tumouricidal activity. In contrast, M2 macrophages are involved in parasite containment and promotion of tissue remodelling, tumour progression and to have immunoregulatory functions.^36^

M1 was observed in exacerbation of lung injury and airway remodelling in allergic asthma via nitric oxide production. The presence of M1 macrophages in tumour microenvironment has been associated with extended survival of certain cancers^3^ also through the production of several angiogenic and lymphangiogenic factors.^37^ M2‐polarized macrophages can be further divided into three subpopulations: M2a, M2b and M2c, according to specific stimulators (cytokines, chemokines). M2a is triggered by IL4 and IL‐13 and positively correlate with the severity of airway inflammation in allergic asthma.^38^ In cancer, a low M1/M2a ratio was associated with poor prognosis in a variety of murine and human malignancies. M2b and M2c are involved in immune regulation, tissue remodelling, angiogenesis and tumour progression. M2b is induced by IgG immunoglobulin complex and lipopolysaccharide (LPS) and are reported in the context of allergy as well as cancer. In allergy, IgG4 can redirect pro‐allergic M2a macrophages to an M2b‐like immunosuppressive phenotype.^39^ This suggests a role of M2b in immune tolerance and so in allergen immunotherapy. On the contrary, in cancer, this phenotype seems to be correlated with disease progression. Recently, high serum level of IgG4 was found in colorectal cancer patients turning M2 macrophages to tolerogenic states favouring cancer environment.^40^ M2c induced by glucocorticoids, TGF‐β and IL‐10 and support induction of Tregs, correlate with tumour progression and poor prognosis.^41^ Tumour‐associated macrophages (TAMs) differentiate from circulating monocytes, enrolled to tumour sites by pro‐inflammatory chemokines (CCL2, CCL3, CCL5, VEGF, colony‐stimulating factors GM‐CSF and M‐CSF).^42^ The prevalence of macrophage phenotype in tumour environment depends on the type of tumour, stage and the place of the tumour. M1/M2 ratio determines the negative prognosis in glioma and breast cancer and the best prognosis in carcinoma of the stomach, colon, prostate and non‐small cell lung.^43^

Immune complexes with an antitumour IgE antibody or crosslinking of surface‐bound IgE can polarize monocytes and macrophages to upregulate CD80 and the pro‐inflammatory mediator TNFα. TNF‐α can then stimulate the production of the macrophage chemoattractant protein 1 (MCP‐1) by both monocytes and tumour cells, and trigger the recruitment of macrophages into tumour lesions and restriction of tumour growth.^44^ In fact, it should be recalled that TNFalfa takes its name from the identification in tumour necrosis. The M2 phenotype predominates in hypoxic areas and seems to have unfavourable effects in tumour growth.

New therapeutic strategies will be available to re‐educate these macrophages promoting the positive effect of M1 phenotype.^45^

### 2.2 Dendritic cells

DCs are ‘skilled’ cells responsible of uptake, proteolytic processing and presentation of antigens to T cells. In an allergic condition, they activate naïve CD4+ T cells to differentiate into Type 2 helper T cell through the production of specific cytokines. Th2 cells and their cytokine production driven by IL‐4 and IL‐13 promote/facilitate the production of allergen‐specific immunoglobulin E (IgE) from B cells**.** Therefore, IgE‐mediated antigen presentation supports DC‐based immunity rather than leading to DC‐mediated tolerance.

On the contrary, activated DCs are converted into tolerogenic phenotypes in the tumour microenvironment, where they promote Tregs (and not T‐effector cells), with the production of TGFβ and IL‐10 as an escape mechanism from immune clearance.^1^, ^2^ Therefore, allergy and cancer have different dendritic cell phenotypes, prevailing the activated DC in atopic subjects, while the antigen presentation by tolerizing DCs is induced in cancer environment preventing anti‐tumour T‐cell responses. The possibility to drive the activation of effector DCs can be a key to stimulate anti‐tumour immunity by the activation of cytotoxic CD8 + lymphocytes against tumour antigens.^46^

### 2.3 Natural killer cells

NK cells are a component of innate immune system expressing eomesodermin and producing cytotoxic granzymes and perforin.

Although the effect of NK cells in subjects with allergy is inadequately analysed, some reports propose a responsibility for NK cells in allergic patients. Different experimentations confirmed that NK cells participate in Th1 cell expansion, allergen‐specific immune suppression, as well as IgE generation. With respect to non‐allergic subjects, augmented NK cell proliferation has been reported in subjects with allergic rhinitis. Moreover, the cytotoxic effect of NK cells in allergic subjects was also greater with respect to healthy controls, while the presence of NK cell costimulatory and inhibitory receptors in allergic subjects displayed heterogeneity in immune control. NK cells involve skin immune responses to hastens by producing type 1 cytokines. Finally, NK cells isolated from the skin of subjects with allergic contact dermatitis presented specific phenotypes.^47^, ^48^

Their cytotoxic role is important especially in the first phase of cancer immunoediting, known as *‘elimination phase’.* NK cells can kill tumour or virally infected cells without any necessity to be primed and proliferated by the first exposure. This is a promising feature for developing new treatments against cancer. Indeed, the prominent role of NK cells leads to future perspective in immunotherapy consisting in adoptive transfer of allogenic NK cells, use of NK cell lines, genetically modified NK cells and antibody therapies.^49^

### 2.4 Innate lymphoid cells

Innate lymphoid cells (ILCs) that include cytotoxic NK play a significant role in the early defence against infections, allergic inflammation, tissue repair and cancer editing.^50^ They reflect helper T‐cell subsets, but they do not express specific antigen receptors. ILCs are classified into three groups, based on their cytokine production. ILC1s, phenotypically like Th1, are characterized by expression of the transcription factor *T‐bet* and production of IFN‐ϒ, respond to IL‐12, IL‐15 and IL‐18. ILC2s, Th2 cells like, are functionally regulated by the transcription factor GATA‐3, respond to epithelium‐derived cytokines, such as IL‐33, IL‐25, TSLP, eicosanoids and IL1‐β. ILC2s are defined by the production of IL‐4, IL‐5, IL‐9 ed IL‐13. Activated ILC2s participate in both initiation and in enhancement of allergy interacting with other immune cells, as macrophages and DCs. In cancer, the stimulation of ILC2s secreted by macrophages through IL‐33 induces the secretion of IL‐13 and IL‐5, which favour tumour progression. On the other hand, amphiregulin‐stimulated ILC2s can establish an immunosuppressive tumour microenvironment.

ILC3s, resemble Th17 and Th22 cells, are characterized by RORϒt transcription, respond to IL‐1β and IL‐23 and are defined by the production of IL‐17 A ed IL‐22.^51^ Cells of the ILC3 subtype secrete IL‐22 upon IL‐23 stimulation by macrophages and have tumourigenic effects. Furthermore, ILC3 could induce tolerance by increasing IL‐10 and retinoic acid secretion by DCs upon stimulation by microbiota and macrophages or by enabling T‐cell tolerance through the expression of MCH Class II in the absence of costimulatory molecules. Among the ILCs type, ILC3s seems to favour tumour growth and tolerance.^52^

### 2.5 T and B lymphocytes

Th2 cells play an essential role in the induction and maintenance of the allergic inflammatory modulation by the production of IL‐4, IL‐5, IL‐6, IL‐9, IL‐10 and IL‐13. They induce differentiation, activation and in situ survival of eosinophils (through IL‐5), stimulate B‐lymphocytes to produce IgE (through IL‐4 or IL‐13), and favour mast cell and basophil growth (through IL‐4, IL‐9 and IL‐10). Their role in cancer is controversial. It has been observed that the shift in immune response from Th1 to Th2 is characteristic of patients with more aggressive tumours.^53^ In some cancers, including breast, gastric and pancreas Th2 cells and associated cytokines (IL‐4 and IL‐13 and TSLP) contribute to tumour progression.^2^ Thus, IL‐4 and IL‐13 receptors are promising anticancer targets. However, in some types of cancer, the Th2 have a protective role (Hodgkin's lymphoma and colon cancer cells). Th1 and T CD8+ lymphocytes play a significant role in the suppression of cancer cells both directly and through the production of IFN‐y, which mediates the activation of macrophages, the presentation and processing of antigens.^54^

B lymphocytes, stimulated by Th2 cytokines, produce IgE which are essential in the development of allergy. On the contrary, B regulatory cells, parallel to Treg cells, inhibit allergic sensitization.^55^ Bregs are a major source of IgG4 that have a positive effect in allergy as immunotolerance but not in cancer where they promote disease progression. B cells are present in many solid tumours (melanoma, colorectal and no small cell lung) and are associated with an improved prognosis. Particularly, B cells associated with T CD8+ cells suggest a cooperation between T and B lymphocytes in inducing an effective anti‐tumour immune reaction.^3^

Tumour‐infiltrating B cells (TiBCs) are associated with improved prognosis in different cancer types. They can mediate immune response against tumours by several mechanisms: production of antibodies, direct cytotoxicity, immunomodulation and promotion of antigen presentation.^56^

Different types of T and B cells are the T and B regulatory cells (Tregs and Bregs). Treg cells favour tumour progression by promoting immune tolerance. They produce inhibitory cytokines (IL‐10, TGF‐β and IL‐35), direct target of DCs via inhibitory PD‐1 and CTLA‐4 cell surface checkpoint molecules and metabolic disruption of effector cells.^57^

While Treg decreases the risk of allergic sensitization and promote immune tolerance, in cancer Tregs contribute to an immunosuppressive tumour microenvironment favouring tumour progression. Tregs are associated with poor prognosis in many cancers, such as ovarian, pancreatic, glioblastoma, lung cancer or melanoma. Cancer immunotherapy targeting Tregs while breaking tumour tolerance can also break tolerance to self, with the alteration of the immune balance and the risk of autoimmunity.^58^, ^59^ Bregs mediate allergen tolerance by IL‐10 mechanism and restrain inflammatory responses. Other mechanisms IL‐10 independent mediated by Bregs are the production of TGFβ, IL‐35, the promotion of T‐cell apoptosis by Fas‐Fas ligand or granzyme pathways, the production of inhibitory IgG4.

B regulatory cells are poorly investigated in cancer. They have an emerging role in cancer, promoting immune tolerance and potentiating Treg responses, negatively regulating anti‐tumour immunity and promoting cancer growth. Bregs should be considered in developing new strategies for cancer therapy, considering the immunosuppressive role of IgG4.

In the future, human Bregs can be eliminated by chimeric antigen receptor T cell. In addition, while lack of Tregs causes severe autoimmune reaction Bregs does not, and it should be considered the ideal treatment.^60^, ^61^

### 2.6 T follicular helper cells

T follicular helper cells (T_FH_) are the primary T‐cell subset responsible for directing the affinity, longevity and isotype of antibodies produced by B cells; they are responsible for IgE responses. Different T_FH_ cell subsets establish the outcome of antibody response. T_FH1_ cells elicited by type 1 immune response (bacterial or viral infections) promote pathogen neutralizing IgGs via production of IL‐21 and interferon‐ϒ (IFN‐ϒ), with limited IL‐4 production. During the type 2 immune responses to helminth infection, IL‐4– and IL‐21–producing T_FH2_ cells are induced, resulting in the production of IgG and low‐affinity IgE antibodies but not anaphylaxis. In contrast, T_FH13_ cells are induced during allergic conditions and are necessary for the generation of high‐affinity IgE, which results in anaphylactic responses. In allergy field, they represent a possible target for diagnosis and therapy.^62^ Indeed, T_FH_ cells are critical regulators of immune responses in several human malignancies such as hepatocellular, breast cancer, ovarian cancer and non‐small lung cancer (NSCLC). According to current understanding, an optimal number of T_FH_ cells are essential for successful antibody secretion by B cells in germinal centre (GC). Decreased percentage of T_FH_ cells diminishes B‐cell recycling, whereas excessive T_FH_ cells drive aberrant B cell production but compromise their affinity and specificity.^63^, ^64^

In breast cancer, T_FH_ cells distinguish extensive immune infiltrates, principally located in tertiary lymphoid structures (TLS) that have been previously identified in lung and colorectal cancers, and their presence has been linked with positive patient prognosis. T_FH_ signature, signifying organized antitumour immunity, robustly predicted survival or pre‐operative response to chemotherapy.^65^

The presence of CD4+ T_FH_ cells in breast cancer represents a positive prognostic factor. Gu‐Trantien et al. show in their study that T_FH_ cells are an important constituent of TLS in breast cancer. Instead, in gastric cancer Th1‐follicular helper T cells seem to contribute to inflammation and tumour development.^66^ In ovarian cancer T_FH_ cells PD‐1+ presented higher IL‐21 and IL‐10 secretion and stronger proliferation from non‐cancer (NC) controls.^67^ T follicular helper cells and T follicular regulatory (T_FR_) cells are identified as the new subset of immune cells. Taken together, there was a significantly higher percentage of T_FH_ and T_FR_ cells in NSCLC patients.

Data showed that the frequency of T_FH_ cells in peripheral blood was significantly lower in NSCLC patients than in healthy controls. In both primary and metastatic tumours, infiltration of T_FH_ cells was observed, suggesting that they participated in the antitumour immunity of NSCLC patients. Compared to other T‐cell subsets, the T_FH_ cells from the peripheral blood and the resected tumours of NSCLC patients presented elevated apoptosis and reduced proliferation capacity. The T_FH_ cells from NSCLC patients were also less effective at downregulating IgD and upregulating CD27 expression in naive B cells, and induced less IgM, IgG and IgA secretion, than those from healthy controls. Overall, it can be concluded that T_FH_ cells were involved in the antitumour immunity and were associated with better clinical outcomes but suffered strong immunosuppression in NSCLC.

Enhancing the T_FH_ cell activity, therefore, represents a potential therapeutic strategy in NSCLC.^68^

### 2.7 Mucosal‐associated invariant T cells

Numerous investigations on mucosal‐associated invariant T (MAIT) cells have recognized correlations between MAIT cell number and clinical prognosis in different malignancies. MAIT cells are a group of innate‐like T cells that present a T‐cell antigen receptor (TCR) that identifies microbially originated non‐peptide antigens presented by the MHC class‐1 such as molecule, MR1. Tumour subjects present augmented rates of MAIT cells within cancerous tissues with respect to controls, with a parallel reduction in the blood circulation, proposing addition suggesting accretion of MAIT cells into the tumour site. However, different analyses performed to study cancer metastases stated reduced rates of MAIT cells. This could suggest that there are dissimilarities in MAIT cell presence of primary tumour with respect to metastatic sites. It is also imaginable possible that MAIT cells in the metastases are ineffectual.

As far their effects, tumour‐infiltrating MAIT cells show reduced IFN‐γ and TNF‐α generation, with augmented IL‐17 production, a cytokine able to increase to tumour angiogenesis.^69^

As for the effects of these cells on allergic diseases, several findings have demonstrated that MAIT cells may be involved in asthma. A study reported that augmented rates of MAIT cells in children was correlated with a decreased risk of asthma in subsequent years, while adult asthma patients presented reduced amounts of MAIT cells in blood, bronchoalveolar lavage and sputum. Moreover, Ye et al. stated an effect for MAIT cells in reducing ILC2 responses and containing allergic airway inflammation. They proved that Mr1^−/−^ mice that were deficient in MAIT cells had intensified ILC2 responses, augmented airway inflammation in response to Alternaria inhalation. Transfer of MAIT cells reduced ILC2 activities and decreased airway inflammation, probably via an augmented expression of the anti‐inflammatory molecule IL4I1. Finally, MAIT cells were reduced after reiterated allergen exposure. The reduction of MAIT cells in asthma patients might participate in an augmented inflammatory response to allergens.^70^

### 2.8 Mast cells

Mast cells are the main effector cells of allergy. They produce many cytokines, chemokines, growth factors (IL‐1, IL‐3, IL‐4, IL‐5, IL‐6, IL‐8, IL‐10, IL‐13, IL‐16, TNF‐α, MCP‐1, VEGF and NGF) and eicosanoids (prostaglandin D_2_ and cysteinyl leukotrienes). These cells also release preformed mediators, such as histamine, heparin and neutral proteases (chymase and tryptase) when activated by both FcɛRI dependent and FcɛRI independent stimuli. Mast cells can also be activated by other stimuli (CD30 ligand, IL1 and TLR‐2), to release selected cytokines and chemokines without degranulation. They have a leading role not only as effector cells of an IgE‐dependent reaction, but through released proteins, participate in the initiation of the allergic immune response, providing signals inducing IgE synthesis by B‐lymphocytes and Th2 lymphocyte differentiation. Beyond allergy, mast cells have critical proinflammatory activity, as well as potential immunoregulatory roles, in various immune and inflammatory disorders. They are involved in host defence mechanisms against parasitic infestations, tissue repair and angiogenesis. Human mast cells produce VEGFs and have VEGF receptors on their surface; therefore, they are both a source and a target of VEGF. In fact, targeting mast cells and their angiogenic factors could be a strategy to block inflammatory and neoplastic angiogenesis.^71^, ^72^

In the tumour microenvironment (TME), multiple stimuli activate mast cells including anti‐tumour antibodies, hypoxia, alarmins, cytokines and chemokines.^73^

Stem cell factor (SCF) seems to be one of the most important substances attracting mast cells into TME where they secrete pro‐angiogenic factors, which promote tumour vascularization and invasiveness. Products attracting mast cells in TME includes angiopoietins and several chemokines (CXCL8, CXCL2, CXCL1, and CXCL10, PGE_2_, TSLP and osteopontin). Furthermore, SCF stimulates mast cells to produce matrix metalloprotease‐9 (MMP‐9) that facilitates the recruitment of other mast cells to the tumour and increases tumour‐derived SCF production in an amplification feedback loop. Mast cells may also suppress the development of protective antitumour immune responses by promoting regulatory T‐cell‐mediated suppression in the tumour microenvironment.^1^, ^2^, ^3^

Thus, mast cells can have both tumour‐promoting and tumour‐inhibiting immunoregulatory effects. It seems that their role depends on microlocalization, stage of tumour and on mast cells density in intratumourally and/or peritumourally.^1^, ^2^, ^3^

In TME it is possible to distinguish anti‐tumourigenic mast cells (MC‐1) and pro‐tumourigenic mast cells (MC‐2). MC‐1 can exert anti‐tumourigenic effect through cytotoxic action (ROS, TNF‐α and granzymes), production of IL‐9 that inhibit tumour cell engraftment, release of histamine promotes dendritic cell (DC) maturation and inhibits tumour growth; tryptase can be taken up into the nucleus of human melanoma cells causing truncation of histones and inhibition of cell proliferation. In addition, human mast cells also can release lymphangiogenic factors (VEGF‐C and VEGF‐D). On the contrary, mast cells can exert pro‐tumourigenic effect through angiogenic molecules (VEGF‐A, VEGF‐B, FGF‐2 and Tryptase).

MMP‐9 can induce degradation of the extracellular matrix, cancer cell invasion and metastasis. TGF‐β, CXCL8 and TNF‐α can induce epithelial to mesenchymal transition. Proinflammatory cytokines such as IL‐1β and IL‐6 can contribute to chronic inflammation in tumour microenvironment. IL‐13 favours M2 polarization of tumour‐associated macrophages. Adenosine can be released by activated mast cells and potentiates the release of angiogenic and lymphangiogenic factors from human mast cells.^74^

In certain neoplasia (e.g., thyroid, gastric, bladder, pancreas, Hodgkin's and non‐Hodgkin's lymphoma) mast cells play a pro‐tumourigenic role, in others (e.g., breast cancer) a protective role, whereas in yet others they are apparently innocent bystanders. In stage I NSCLC, but not in stage II, peritumoural, but not intra‐tumoural mast cell density is an independent favourable prognostic factor; mast cells were pro‐tumourigenic in the initial stages of prostate cancer but not in the later stages; in perilesional stroma of melanoma play a protective role. In pancreatic ductal adenocarcinoma, mast cell density in the intratumoural border zone, but not the peritumoural or the intratumoural centre zone, was associated with a worse prognosis. In prostate cancer, high intratumoural mast cell density was initially associated with good prognosis. Generally, it was reported that intratumoural mast cells inhibited tumour growth, whereas peritumoural mast cells stimulated human prostate cancer.^75^

These findings suggest that the microlocalization of mast cells should be investigated in various stages of clinical and experimental tumours. Last but not the least, the protumourigenic activities of mast cells can be subverted by targeting cells to promote tumour destruction. Furthermore, mast cells cause tumour cells death, in an in vitro lymphoma model, when incubated with an anti‐CD20 IgE antibody.^76^ These findings represent the potential to deviate the response of these cells against cancer through immunotherapies.

### 2.9 Eosinophils

Eosinophils are multifunctional cells with pleiotropic functions. They are implicated in protection against parasitic infections, allergic reactions and chronic inflammatory diseases. Activated eosinophils release cytotoxic proteins (e.g., ECP, MBP, EPX and EDN), growth factors, cytokines, chemokines and lipid mediators contributing to inflammation.

Recent experimental studies show that eosinophils are not homogenous population and like other immune cells have different phenotypes. It is possible to distinguish inflammatory eosinophils (iEos) and regulatory eosinophils (rEos) based on their surface marker expression.^77^ Eosinophils are shaped by the morphogenetic plasticity of their environment, so different subtypes of eosinophils depend on maturation phase, organ location, morphogenetic activity of the tissue and location within tissue. Murine eosinophil sub‐phenotypes as falling within one of these four tissue‐based categories: (1) EoP. Immature eosinophils recruited as precursors or undergoing in situ haematopoiesis. (2) Steady state. True tissue residents in morphogenetically quiescent tissues. (3) Type 1. Typically, interstitial (stromal in general) in acute inflammatory, innate defence and transient morphogenetic contexts. (4) Type 2. Eosinophils associated with a Type 2 immune response are typically found in epithelial contexts. Different eosinophil subsets may co‐exist and perform different functions in the same tissue. For example, based on their location and association with immune and morphogenetic environments, Type 1 eosinophils may preferentially interact with fibroblasts and assist in building ECM scaffolds, while Type 2 eosinophils may directly interact with the epithelium and participate in scaffold removal and resolution of repair. Eosinophils have also been reported to participate in the control of tumour growth and the formation of metastasis.^78^

In most epidemiological and clinical studies, eosinophils demonstrate ‘tumouricidal’ action mediated by α‐defensins, TNF‐α, granzymes A and IL‐18.^79^ Moreover, eosinophils might support antitumour immune responses indirectly, for example, by facilitating T‐cell migration into tumours.

Tumour cells themselves can attract eosinophils by producing CCL1 and stimulating eosinophils to secrete IL‐8 that facilitates eosinophil–cancer cell interaction leading to tumour cell death. In allergic patients, they show a greater cytotoxic action, and this suggests that the ‘‘allergy state’’ promotes anticancer processes. In future, eosinophils can be activated by immunotherapy such as checkpoint inhibitors or GM‐CSF‐based vaccines, or by adoptive transfer of these cells in an appropriate setting.

In several neoplasias (e.g., melanoma, gastric, colorectal, oral and prostate cancer), eosinophils play an anti‐tumourigenic role, in others (e.g., Hodgkin's lymphoma, cervical carcinoma) have been linked to poor prognosis, whereas in yet others, they are apparently innocent bystanders. The role of eosinophils and their mediators appears to be cancer dependent. The microlocalization (e.g., peritumoural vs. intratumoural) of eosinophils could be another important aspect in the initiation/progression of solid and haematological tumours.^80^

Recently*,* Holland et al. have demonstrated that the increased expression of dipeptidyl peptidase 4 (also known as CD26) has been observed in mouse and human tumours and is associated with worse survival. On the contrary, the inhibition of CD26 can improve antitumour immune response by enhancing the effect of eosinophils through IL‐33‐depended eosinophil‐mediated control of tumour growth. IL‐33, a tumour‐derived alarmin, in solid tumour induces eosinophil migration and promotes CCL11‐mediated eosinophil infiltration and degranulation, which in turn leads to tumour cell cytotoxicity and reduced tumour growth. In addition, it has been demonstrated that combined immunotherapy using checkpoint blockade in the presence of CD26i inhibits tumour growth.^81^

### 2.10 Epithelial cells

Epithelial barrier has an essential role to balance immune response. Epithelial cells surface includes the high and low‐affinity IgE Fc receptors facilitating antigen passage and direct antigen presentation. Epithelium constitutes a source of cytokine and contributes to modulation of the immune response both in allergy and in cancer. Intestinal epithelial cells of extracellular vesicles contribute to innate immunosuppression that generate oral tolerance or cancer progression.^3^, ^82^

Role of different immune cells in cancer is listed in Table 1.

**TABLE 1:** Role of immune cells in cancer

Cells	Tumour‐promoting effect	Tumour‐inhibiting effect	Reference
Macrophages	M2 phenotype (M2a, M2b, M2c)	M1 phenotype	^38^, ^39^, ^40^, ^41^, ^42^
Dendritic cells (DC)	DCs tolerogenic phenotype	DCs activated phenotype	^46^
Natural killer (NK)		Cytotoxic effect (granzymes, perforins)	^49^
Innate lymphoid cells	IL‐25 stimulated (IL‐13, IL‐5) ILC2 ILC3	IL‐33 and amphiregulin stimulated ILC2	^50,52^
Lymphocytes	Th2 (IL‐4, IL‐13, TSLP)	Th2 (Hodgkin's lymphoma, colon cancer) Th1	^2^, ^3^, ^53^, ^54^, ^56^, ^58^, ^61^
	Treg lymphocytes Breg lymphocytes	TCD8+ (IFN‐ϒ)	
	Lymphocytes	B Lymphocytes	
T follicular helper cells (TFH)	↓ TFH	↑TFH (antibody secretion by B cells; breast cancer, colorectal cancer, NSCLC)	^63^, ^64^, ^65^, ^66^, ^68^
	TFR (T follicular regulatory cells)		
	Th1 follicular helper (gastric cancer)		
Mast cells	Pro‐tumourigenic mast cells (MC‐2) trough angio‐genic factors (VEGF‐A, VEGF‐B, FGF‐2, tryp‐ tase	Antitumourigenic‐ mast cells (MC‐1) trough cytotoxic action (ROS, TNF‐α, granzymes)	^3^, ^73^, ^74^
Eosinophils	rEOS, Type1 eosinophils	Eosinophils (α‐defensins, TNF‐α, granzymes A and IL‐18); iEOS	^75^, ^79^

## 3 ROLE OF IMMUNE PROTEINS

### 3.1 IgE

**FIGURE 1:**
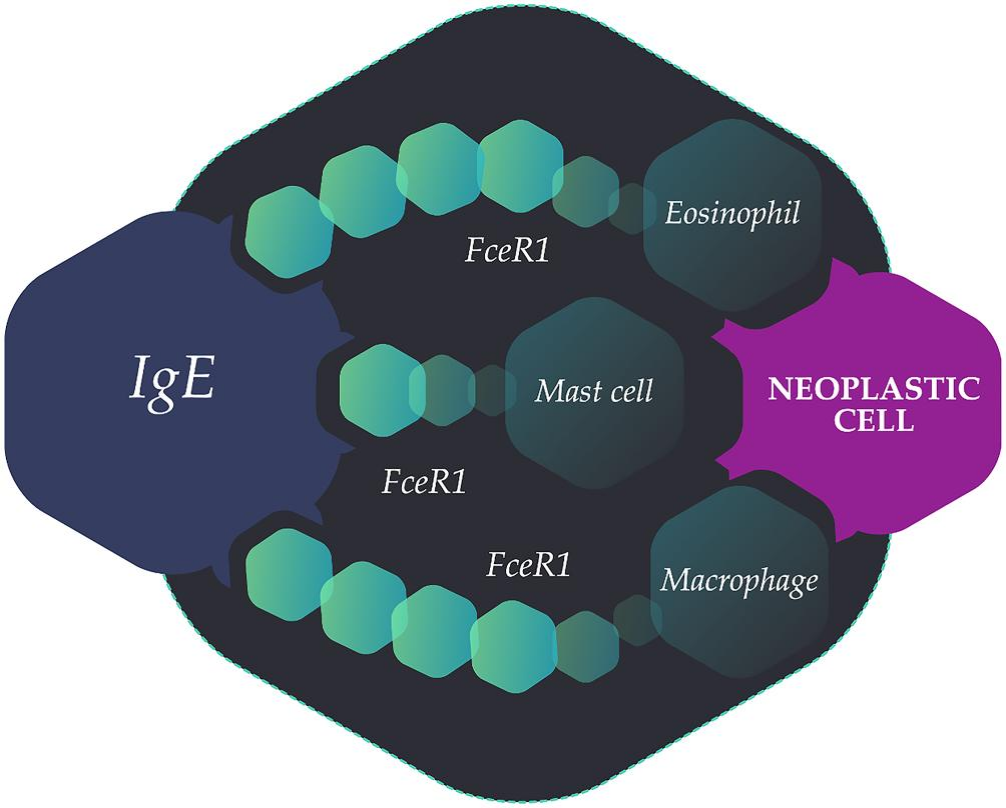
Immunoglobulin E is able to bind Fc receptors on tumour associated effector cells promoting tumouricidal actions

IgE is an evolutionarily conserved member of the Ig family with the highest determined affinity to receptors and antigens among all antibody classes.^83^ IgE is known as a biomarker in atopy, allergy and parasitic infestations. Recently, epidemiological studies in vitro and in vivo indicate that natural IgE has a surveillance function in cancer. IgE antibodies directed against tumour‐associated antigens (TAA) could mediate the cell‐to‐cell association between tumour and effector cells, resulting in antibody‐dependent cellular toxicity (ADCC) and antibody‐dependent cellular phagocytosis (ADCP). The prominent function of IgE able to bind Fc receptor (FcɛRI) on tumour‐associated effector cells, such as eosinophils, mast cells and macrophages, promoting tumouricidal actions and a more favourable prognosis in cancer (Figure 1).

Since the potent anticancer role of IgE antibodies in vitro engineering antibodies were created. The Fc regions of IgE class specific for cancer antigens are designed and tested in vitro and in vivo. Recombinant IgE can be generated by different cloning strategies: classical restriction enzyme‐based cloning, human/mouse chimeric IgEs; in future, fully human IgE antibodies could be generated. Additionally, it should be considered the glycosylated structure of the IgE antibody, which seems to be changed in healthy and in different diseases. Recently, the anti‐HER2/neu, anti‐EGFR IgG1 antibodies trastuzumab and cetuximab have been cloned and engineered recombinantly as humanized and chimeric IgE antibodies^84^ and even anti‐CD20 IgE antibody.^85^

Indeed, recombinant anti‐cancer IgE is under investigation in a human trial (NCT02546921) with promising results [https://clinicaltrials.gov/ct2/show/NCT02546921].

Several epidemiological studies reported an inverse association between atopy and cancer (pancreatic, prostatic, colorectal cancer, brain tumour, melanoma, breast and gynaecological cancers, chronic lymphocytic leukaemia, and multiple myeloma). However, one study of postmenopausal women showed no association between self‐reported environmental allergies and incident myeloid or lymphoid malignancies.^86^, ^87^ These findings suggest that the relationship between atopy and malignancy is complex and probably depends on tumour types and the individual studied populations. Despite some mixed results, a 2016 review tabulating the body of epidemiological evidence of the relationship between atopy and cancer risk since 1995, suggested that atopy was associated with a reduced cancer risk.^88^ Interestingly, IgE deficiency (IgE <2.5 kU/L or IgE <2 kU/L) is associated with a higher risk of malignancies^89^ and *ultra‐low IgE* is a potential novel biomarker in cancer.

### 3.2 IgG4

Allergen immunotherapy aims to achieve immune tolerance to a specific allergen. IgG4 is a marker of immune tolerance supported by IL‐10 and TGF‐β cells, such as T and B regulatory cells. While immune tolerance is a goal in allergy field, in tumour microenvironment represents an antitumour immunity escape. IgG4 might repolarize M2a macrophages to the immunosuppressive phenotype M2b, which could be responsible for increased IL‐10 secretion. IgG4 is expressed in tissues from patients with malignancies such as melanoma, in whom it can impair antitumour immunity and correlates with shorter survival and disease progression.^90^ There is also increasing evidence to support positive correlations between IgG4‐related diseases, such as sclerosing cholangitis associated with autoimmune pancreatitis, with enhanced cancer risk. Elevated IgG4 has been detected in extrahepatic cholangiocarcinoma, colorectal cancer, pancreatic cancer, melanoma and glioblastoma.^91^

### 3.3 Free light chains

FLCs are a product of immunoglobulin heavy chains. FLC levels have been measured in allergic diseases (asthma, rhinitis), chronic obstructive pulmonary disease (COPD), rheumatoid arthritis, multiple sclerosis, diabetes, inflammatory bowel disease and cancer.^92^ In allergy fields, immunoglobulin FLCs induced non‐IgE‐mediated mast cell activation and release of mediators without degranulation.^93^

In the latter, FLC seems to contribute to cancer progression through antigen‐specific mast cell activation and reduce neutrophil apoptosis and stimulate the release of pro‐tumourigenic IL‐8. FLC was found to be a biomarker for poor prognosis in basal‐like breast cancer; it was demonstrated that FLC stimulated tumourigenesis through mast cell activation in melanoma model.^94^

### 3.4 Regulatory cytokines (IL‐10, TGF‐β) and chemokines (CCCL1, CCCL5)

IL‐10 and TGF‐β are regulatory cytokines with a pivotal role in immune tolerance in allergy field whereas promoting cancer growth and progression. CCCL1 is a chemokine expressed by monocytes and by tolerogenic M2b macrophages subtypes and have a regulatory role. In contrast, depending on the tumour environment, CCCL5 chemokine has demonstrated an antitumour role, in particular, interventions on CCR5 expressed by tumour cells resulted in antitumour effect.^3^

### 3.5 Lipocalins

Lipocalins are innate defence proteins. Lipocalin‐2 (LCN2) is upregulated in various cancer types, while they are decreased in allergic and atopic state and this, also has been proposed as a cancer biomarker.^3^, ^95^

### 3.6 Alarmins (HMGB1, IL1‐α, S100 and IL‐33)

**FIGURE 2:**
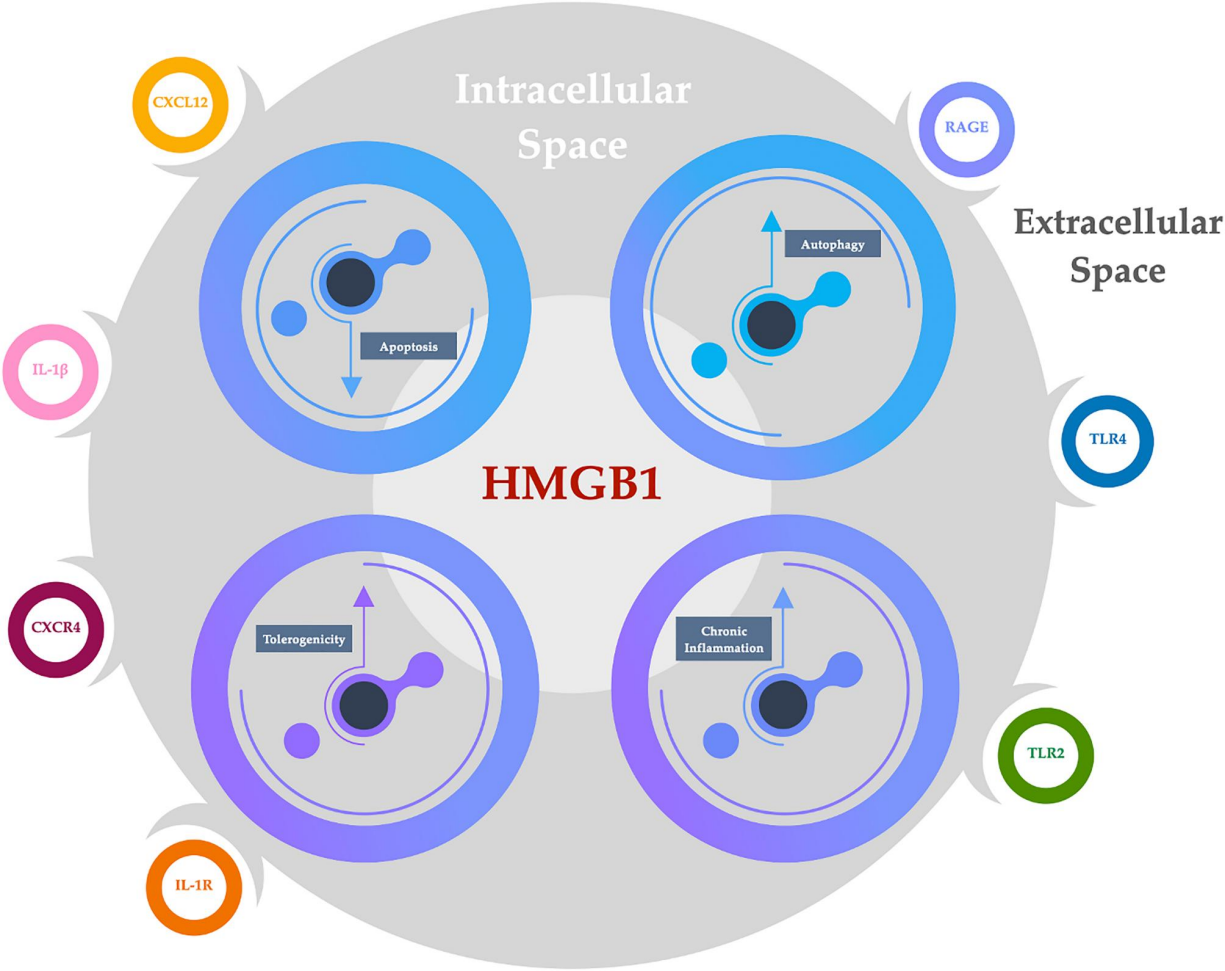
Role of HMGB1 in cancer development

Alarmins are proteins released from host cells after activation or when cells are damaged or died.^96^ They have shown a dual function acting both intracellularly and extracellularly with various biological functions: chemotaxis, direct binding to receptors (e.g., TLR or IL‐1R) and inflammation^97^ (Figure 2). The name ‘Alarmins’ reflects their nature as ‘dangers signals’ crucial for cancer, host defence and inflammatory response. This group of proteins includes the high‐mobility group box 1 protein (HMGB1), interleukin (IL)‐1α, the Ca2+‐binding S100 proteins and IL‐33.

#### 3.6.1 HMGB1

An important class of alarmins is the high mobility group (HMG) proteins consisting of HMGA, HMGB and HMGN families, of which HMGB1 is one the most studied alarmins.^98^ Under resting conditions, HMGB1 is localized in the nucleus, where it contributes to chromatin architecture and gene expression involved in chromosomal DNA repair and genomic stability maintenance. HMGB1 exerts pleiotropic functions, predominantly regulatory function, when it's localized in the nucleus instead of extracellular localization. Its activities from cell localization to extracellular functions are regulated by post‐translational modifications such as acetylation, methylation and phosphorylation.^99^ Monocytes and macrophages hyperacetylate HMGB1 at nuclear localization sites, leading to its cytosolic re‐localization.

This mechanism was recently shown to be mediated by the activation of the JAK/STAT1 (Janus kinase/signal transducer and activator of transcription) pathway.^100^ The redox state of HMGB1 too is believed to orchestrate its extracellular function.^101^ Extracellular HMGB1 mediates inflammation, cell migration, proliferation and differentiation.^102^, ^103^ Cytoplasmic HMGB1 is involved in immune responses by increasing autophagy, inhibiting apoptosis and regulating mitochondrial function^104^ (Figure 2).

In the extracellular space, HMGB1 binds to receptors like receptor for advanced glycation end products (RAGE), TLR4, TLR2, IL‐1R, CXCR4, IL‐1β and CXCL12. In DCs, HMGB1 release and sensing by RAGE was shown to be critical for homing to the lymph nodes and further cross‐activation of T lymphocytes.^105^, ^106^, ^107^ In endothelial cells, HMGB1 was shown to promote the expression of RAGE and surface adhesion proteins (intercellular adhesion molecule 1 (ICAM‐1) and vascular cell adhesion molecule 1 (VCAM‐1) and to induce RAGE‐dependent cytokine production.^108^ Interestingly, yet another receptor of HMGB1, TIM‐3, expressed at the surface of tumour‐associated DCs, was recently shown to compete with nucleic acids for binding to HMGB1, thereby dampening the efficacy of antitumour DNA vaccines or chemotherapy.^109^ Moreover, during apoptotic cell death, ROS production induces the terminal oxidation of HMGB1 that inhibits its proinflammatory function and switches HMGB1 function toward tolerogenicity. Therefore, HMGB1 might be a potential target for the treatment of inflammation.

In allergy field, HMGB1 could aggravate eosinophilic inflammation in the airway of acute allergic asthma through inducing a dominance of Th2‐type response and promoting the neutrophilic inflammation.^110^

For several years, HMBG1 was studied for his role in cancer development and nowadays it is considered one of the most modulators of cancer microenvironment.^111^ On the one hand, HMGB1 can contribute to tumourigenesis. On the other hand, HMGB1 plays a protective role in the suppression of tumour and tumour chemoradiotherapy and immunotherapy. HMGB1 expression increases in many types of cancer, correlates with tumour invasion and metastasis, and relates to worse prognosis.^112^

Combining role with RAGE, contribute to chronic inflammation and tumourigenesis. Further emphasizing the role of the RAGE–HMGB1 axis in cancer progression, blockade of either HMGB1 or RAGE can reduce malignant mesothelioma and glioma tumour growth and metastasis.^113^ In contrast, oxidized HMGB1 promoted cell death and increased the efficiency of chemotherapeutic drugs. In addition, HMGB1 has been implicated in the antitumour immune response induced by radiation therapy or chemotherapy.^114^ In any case, further studies are needed to ulteriorly clarify the role of this alarmin.

#### 3.6.2 IL‐1α

IL‐1α is a dual function cytokine with both nuclear and extracellular functions. As HMGB1, it is involved in inflammation and cancer.^115^ IL‐1α precursor is expressed in the nucleus of non‐hematopoietic cells (epithelial cells of gastrointestinal tract, kidney, liver and skin), as a transcription factor, regulate gene expression and growth and differentiation of cells.^116^

In stimulated cells, IL‐1α is processed by the membrane‐bound protease calpain, a calcium‐dependent cysteine protease, and then released into the extracellular space. Furthermore, to calpain‐ and caspase‐1‐dependent pIL‐1α processing, other proteases, such as granzyme B, elastase or chymase cleave pIL‐1α, producing the mature form of IL‐1α and potentiating its proinflammatory activity.^117^ For instance, in melanoma patients, IL‐1α induced the MyD88‐dependent activation of the NF‐κB and MAPK pathways as well as an increase in ROS production, promotes tumour progression. Additionally, in pancreatic ductal adenocarcinoma, tumour‐associated IL‐1α release, probably through cell damage, can also induce tumour growth. IL‐1α is also implicated in tumour vascularization by promoting the expression of vascular endothelial growth factor (VEGF) in endothelial cells.^118^ Yet, clinical trials are underway using MABp1 antibody to target IL‐1α in the treatment of refractory cancers with metastasis.^119^

In contrast to the role of secreted IL‐1α, intracellular and membrane‐bound IL‐1α activates immune mechanisms that lead to tumour destruction. Indeed, in a large panel of cancer cell lines, pIL‐1α modulates cell cycle and induces apoptosis.^120^ Drugs in clinical use, such as anakinra, give hope for the targeting of IL‐1α in these pathologies. However, the complex multifaceted functions of IL‐1α, sometimes beneficial and at other times deleterious, make IL‐1α a difficult clinical target.

In allergy field, IL‐1alpha administration during sensitization of Th2‐mediated allergic reactions has been shown to suppress the course of disease by shifting the immune response towards Th1.^121^

#### 3.6.3 S100

S100 is part of the S100 family of proteins composed of 25 members with different intracellular and/or extracellular functions. They have a high grade of similarity in sequence and structure, but they are not interchangeable, and they have different biological functions. S100 proteins are crucial proteins for calcium homeostasis and the maintenance of sufficient intracellular concentration of Ca2++ for cell metabolism.^122^

Such as HMB1, IL1‐α and IL‐33 are a dual‐function protein. They are considered intracellular transcriptor factors and when they are released in extracellular space interact with several receptors, the most important are RAGE and TLR4, and mediate proinflammatory state promoting cell migration, proliferation and differentiation. S100 proteins have several physiological functions such as scavenging of ROS and nitric oxide, cytoskeleton assembly, membrane protein recruitment and trafficking, transcriptional regulation and DNA repair, cell differentiation, release of cytokines and antimicrobial agents, muscle cell contractility, cell growth and migration, apoptosis^123^, ^124^, ^125^

S100A8/A9, S100A12 and S100 B are even considered biomarkers of specific diseases, such as cancer, atherosclerosis and stroke.^126^ S100 proteins lack a signal peptide for secretion via the conventional Golgi mediated pathway, and their secretion can occur passively upon cell necrosis or actively after cell activation. S100A8/A9 are extremely sensitive to oxidation and their redox state acts as a molecular switch from a proinflammatory function (reduced) to a protective wound‐healing and antioxidant function (oxidised).^127^

In contrast, oxidation of S100B was shown to be necessary for binding to RAGE and the subsequent increase in the expression of the angiogenic factor VEGF, an important player in the development of macular degeneration.^128^

Innate cytokines such as IL‐1, IL‐33 and thymic stromal lymphopoietin (TSLP), as well as the alarmins HMGB1 and S100 proteins programme DCs to mount Th2‐cell‐mediated immunity and stimulate ILC2, basophil and mast cell function.^129^

Like other alarmins mentioned above, S100 proteins have a significant role in cancer development promoting cell proliferation, metastasis, angiogenesis and immune evasion. However, they have different profile roles depending on type and stage of tumour.^130^, ^131^, ^132^

The dysregulation of S100 protein expression is a common occurrence in many human cancers. Inhibitors directly targeting two family members, S100 B and S100A9, are in clinical trials for melanoma and prostate cancer, respectively. S100 proteins expression are also linked with drug resistance and are involved in chemotherapy response. Every cancer has a specific S100 expression profile. S100 proteins are widely studied in breast cancer, lung cancer, melanoma, ovarian cancer, colorectal and pancreatic cancer, and they represent a source for therapeutic opportunities.^133^ Potential S100 inhibitors are classified in small molecules inhibitors, neutralizing antibodies and miRNA mimics. Small molecules inhibitors inhibit transcription of S100A4, S100A9, S100A10, S100B and S100P. Also, several studies with antibodies neutralizing S100A4, S100A7, S100A8/S100A9 and S100P are in preclinical phase. Several miRNAs were introduced to target the expression of S100 proteins. For instance, miR‐187‐3p and miR‐149‐3p were found to downregulate S100A4 expression.^134^ Role of different S100 proteins is listed in Table 2.

**TABLE 2:** Role of different S100 proteins in cancer

S100 protein	Type of cancer	Tumour‐promoting effect	Tumour‐inhibiting effect	Reference
S100A2	Oral cancer		X	^130^, ^131^, ^132^, ^133^, ^134^
S100A1, S100A2, S100A3, S100A4, S100A6, S100A7, S100A8/S100A9, S100A10, S10 G	Lung cancer	X		^130^, ^131^, ^132^, ^133^, ^134^
S100 B			X	^130^, ^131^, ^132^, ^133^, ^134^
S100A4, S100A7, S100A8, S100A9, S100A11, S100P	Breast cancer	X		^130^, ^131^, ^132^, ^133^, ^134^
S100A1, S100A6			X	^130^, ^131^, ^132^, ^133^, ^134^
S100A4, S100A8/A9,	Melanoma	X		^130^, ^131^, ^132^, ^133^, ^134^
S100 B			X	^130^, ^131^, ^132^, ^133^, ^134^
S100A2, S100A10, S100A11, S100A15, S100A16, S100 B	Ovarian cancer	X		^130^, ^131^, ^132^, ^133^, ^134^
S100A1, S100A3, S100A5, S100A6, S100A13, S100 G, S100Z				^130^, ^131^, ^132^, ^133^, ^134^
S100A4, S100A8/S100A9, S100P	Colorectal cancer	X		^130^, ^131^, ^132^, ^133^, ^134^
S100A2, S100A6, S100A11, S100A4, S100A8/S100A9	Pancreatic cancer	X		^133^

#### 3.6.4 IL‐33

IL‐33 is IL‐1 family member of cytokines exerting pleiotropic functions.^135^ IL‐33 is an ‘‘alarmin’’ that acts considered both as a nuclear factor and a cytokine. During tissue damage, necrosis or injury is released into extracellular space where bind is receptor suppression of tumourigenicity 2 (ST2) express on the membrane of target cells and activate an inflammatory cascade, predominantly T Helper 2 immune response. STL2 is expressed in fibroblasts, mast cells, eosinophils, Th2 lymphocytes, DCs, basophils, NK, macrophages, epithelial cells. IL‐33/ST2 can stimulate an atypical TH2 response through production of IL‐5 and IL‐13 by ILC2 and TH2 cells, activation of NK, NKT and TH1 cells with production of IFN‐ϒ, CD107a exposure and IFN‐ϒ production by CD8 activated, stimulation of T and B reg cells, release of inflammatory cytokines (IL‐1β, IL‐6, TNFβα) by DCs, macrophages and mast cells, M2 macrophage polarization, degranulation of mast cells, basophils and eosinophils, neutrophil migration and activation of DC and eosinophils.^136^

This pleiotropic nature of IL‐33 explain why IL‐33 has been implicated in a wide variety of non‐allergic diseases, including infectious diseases (fungal, helminth, protozoa, bacterial and viral infection), cardiovascular diseases, COPD, fibrotic diseases, musculoskeletal diseases, inflammatory bowel diseases, diseases of the central nervous system (Alzheimer), graft versus host disease (GVHD), obesity, diabetes and cancer. IL‐33 binding receptor ST2 activate Th2 pathway, inducing mast cell degranulation, producing IL‐1β, IL‐3, IL‐6, TNF, CXCL2, CCL2, CCL3, PGD2, LTB4, IL‐5, IL‐13, CCL5, CCL17, CCL‐24. IL‐33 is involved in allergic diseases such as asthma, anaphylaxis, atopic dermatitis and allergic rhinitis.

IL‐33 can have opposite functions in cancer field, promoting or dampening tumour immunity, depending on the tumour type, site of expression and local concentration.^137^

It is known that chronic inflammation contributes to tumourigenesis. Recent findings have revealed an important contribution of IL‐33 to several cancers, where it may exert pro and less frequently anti‐tumourigenic functions. Targeting the IL‐33 pathway represents a potential for cancer therapy.^138^

However, it is now clear that the action of IL‐33 is not limited to the activation of type‐2 immune responses. Indeed, recent studies have revealed important roles of IL‐33 in the activation of immune cells involved in type‐1 immunity, such as Th1 cells, NK cells, CD8+ T cells, neutrophils, macrophages, B cells and NKT cells. Several studies suggested that IL‐33 could be considered as a tumour biomarker; IL‐33/ST2 signalling have a pro‐tumourigenic role in head and neck squamous cancer, breast cancer, non‐small‐cell lung cancer, cholangiocarcinoma, gastric cancer and myeloproliferative neoplasm. On the contrary, IL‐33 has anti‐tumourigenic role in hepatocellular carcinoma and colorectal cancer. In the latter, several studies demonstrate also a pro‐tumourigenic role.^139^

The combination of PD‐1 checkpoint blockade with IL‐33 prolonged mice survival and induced leukaemia regression. This is important evidence for combining IL‐33 with immunotherapy targeting immune checkpoint inhibitors.^140^

IL‐33 promotes IgA production, preventing microbial dysbiosis and IL‐1 dependent inflammation in the intestine. However, it is currently known that manipulation of microbiota may represent a therapeutic strategy for the treatment of colorectal cancer, independently of IL‐33.^141^ ^,^ ^142^

Finally, IL‐33 has an ambiguous role preponderant on tumourigenic implications in tumour microenvironments and this implication can be useful to adopt anti‐IL33 therapy.^143^

IL‐33 represents a promising immune adjuvant for vaccine therapy, tumour biomarker and therapeutic target. Concluding, IL‐33 has an emergent significant role in cancer.

The controversial role depends on tumour microenvironment and type of tumour. Further studies are needed to elucidate the role of this pathway and the probable role as a biomarker predictive of cancer progression and patient survival. Table 3 summarizes the role of immune proteins in cancer.

**TABLE 3:** Role of immune proteins in cancer

Immune proteins	Tumour‐promoting effect	Tumour‐inhibiting effect	Reference
IgE antibodies	↓IgE antibody ultra‐low IgE	IgE antibody against TAA; ADCC, ADCP	^89^, ^90^
IgG4 antibodies	IgG4 (IL‐10, TGF‐β, Treg e Breg) IgG4 (M2a◊M2b)		^3^, ^89^, ^90^
Free‐light chains (FLCs)	↓neutrophil apoptosis, pro‐tumourigenicIL‐8, mast cell activation		^92^
Regulatory cytokines (IL‐10, TGF‐β)	IL‐10, TGF‐β		^3^
Chemokines (CCCL1, CCCL5)	CCCL1	CCCL5	^3^
Lipocalins	Lipocalin‐2 (LCN2)		^3^, ^95^
Alarmins (HMGB1, IL1‐α, S100, IL‐33)	↑ HMGB1‐RAGE	↓HMGB1	^110^, ^111^, ^112^, ^113^
	Extracellular IL1‐α	IL1‐α intracellular and membrane‐bound	^119^, ^120^
	S100 proteins (S100A8/A9, S100A12, S100 B)	IL‐33 (in hepatocellular carcinoma, colorectal cancer	^130^, ^131^, ^132^, ^133^
	Extracellular IL‐33		^138^, ^139^, ^140^
RAGE signalling	↑RAGE signalling (DAMPS, AGEs, HMGB1, S100s, DNA, RNA)	↓ RAGE signalling	^147^, ^148^, ^149^, ^150^

### 3.7 RAGE signalling

The receptor for advanced glycation end‐products (RAGE) is a transmembrane protein belonging to the immunoglobulin (Ig) superfamily of receptors.^144^, ^145^ It is considered a multiligand pattern recognition receptor implicated in chronic inflammation states and it is a key regulator of the innate immune response. RAGE binds and mediates the cellular.

Response to a range of damage‐associated molecular pattern molecules (DAMPs) including advanced glycation end‐products (AGEs) that underlie diabetic complications, HMGB1, S100s and DNA. Under normal conditions, RAGE is expressed at low level, but it results up‐regulated under chronic inflammation such as diabetes, cardiovascular diseases, cancer progression and metastasis.^146^ RAGE is composed of an extracellular region containing three immunoglobulin (Ig) domains, a single transmembrane domain, and an intracellular cytoplasmic domain. The extracellular domain binds the majority of ligands as AGEs, several S100 proteins (S100A4, S100A6, S100A7, S100A8, S100A9, S100A8/9, S100A12, S100B and S100P), HMGB, nucleic acids (DNA, RNA). Extracellular ligands are bacterial LPS, dietary AGEs. As reviewed below, non‐AGE ligand HMGB1 and S100 proteins bind RAGE with finally activation of intracellular signalling resulting in changes gene expression and altered cellular functions including migration, survival, inflammation, and up‐regulation of RAGE expression itself. This important mechanism was a key to develop therapy blocking RAGE signals. Small‐molecule RAGE inhibitors are under pre‐clinical investigations.^147^ Current experimental data indicates that RAGE is a pivotal mediator of type 2 inflammatory reactions which drive the development of T2 high allergic diseases. Clinical studies demonstrate that increased RAGE ligands and signalling strongly correlate with asthma severity, especially in severe neutrophilic asthma. These findings indicate a possible role as a biomarker of disease and potential therapeutic strategies.^148^

RAGE has been implicated in the pathogenesis of a variety of cancer types including breast, glioma, bladder, melanoma, liver, pancreatic, prostate, colorectal, gastric and lung.^149^

Increased RAGE protein expression has been associated with increased tumour histological grade and poorer outcomes in a similar set of cancers.^150^ RAGE alters properties associated with the malignant process, including increased cell migration and invasion, proliferation and resistance to apoptosis. Blocking RAGE signalling in cancer cells reduces tumour growth both in vitro and in *murine models*, and therefore represents an attractive therapeutic target in cancer. Importantly, RAGE inhibition and gene knockout have been shown to impair tumour metastasis revealing a major new target for treating metastatic diseases. However, studies are required to translate these promising findings to human clinical trials now that small‐molecule inhibitors of RAGE have been shown to be well tolerated in humans.

## 4 MiRNA, ALLERGY AND CANCER

miRNAs are short non‐coding RNAs that mediate sequence‐specific repression of target messenger RNAs (mRNAs) inhibiting gene expression at the post‐transcriptional level.^151^

They ensure the normal function of cells and are involved in fundamental biological processes such as development, proliferation, apoptosis, tumourigenesis and immune reactions.^152^

A single miRNA can have multiple mRNA targets, several miRNAs can regulate a single mRNA and the altered expression of a single miRNA can change inflammatory response and cause disease. That is why miRNA represents an attractive therapeutic target, especially for cancer. For example, specific miRNAs that regulate type 2‐mediated immunosuppression in the tumour microenvironment might be altered to induce tumour surveillance and identification of miRNAs involved in allergic sensitization and maintenance of chronic inflammation might be utilised in novel prevention or treatment strategies of allergic diseases in the future. Generally, miRNAs have been classified either as oncogenic (e.g., miR‐155, miR‐17‐5p or miR‐21) or having a tumour suppressor role (e.g., miR‐34, miR‐15a and let‐7).^153^, ^154^, ^155^, ^156^, ^157^, ^158^, ^159^, ^160^, ^161^, ^162^, ^163^, ^164^, ^165^, ^166^, ^167^, ^168^, ^169^, ^170^, ^171^

miRNAs play essential roles in the regulation of carcinogenesis and immune response. Besides, several studies have demonstrated that ROS can regulate miRNA biogenesis, transcription factors and epigenetic changes. On the other hand, miRNAs may, in turn, modulate the redox signalling pathways, altering their integrity, stability and functionality, thus contributing to the pathogenesis of multiple diseases. Both ROS and miRNAs have been identified to be important regulators and potential therapeutic targets in cancers.^172^

The regulation of the immune system by miRNAs in cancer includes Type 2 immune reactions and the involvement of miRNAs in the response initiated by allergens, parasites or other environmental factors are just emerging.^173^

The major functions of miRNAs mediating the immune response are shown in Table 4.

**TABLE 4:** Immune actions of MiRNA

MiRNA	Immune functions related	Reference
miR‐221	Release of IL‐6, proliferation	^154^, ^155^, ^159^
	IgE‐mediated mast cells degranulation	
miR‐19a	Target of TGFβ‐receptor	^154^
miR‐34/449	Increase IL‐13	^155^
miR‐375	Increase IL‐13; amplification Th2 response	^156^
miR‐155	ILC2‐mediated inflammation; regulation of B cells; differentiation CD4+→Th1, Th2	^158^, ^159^
	Favour M1 macrophages phenotype; stimulate dendritic cells; stimulate mast cells;	
miR‐21a, miR‐98, miR‐155 (high levels)	Activation of ILC2 and TH2	^159^, ^160^, ^161^
Let‐7c, miR‐151, miR‐203 (low levels)	Activation of ILC2 and TH2	^159^, ^160^, ^161^
miR‐17‐92 cluster family miR‐19a (high levels)	Negative regulation of tumour necrosis factor alpha‐induced protein 3 (TNFAIP3)	^154^
miR‐125b‐5p, miR‐199b and miR‐378‐3p (high levels)	Favour M2 macrophages inhibition of IL‐5 and IL‐13	^158^
miR‐193b, miR‐342‐3p (induction)	Stimulated by IL‐4 in macrophages	^158^
miR‐99b, miR‐125a‐5p (repression)	Stimulated by IL‐4 in macrophages	^158^
Mir‐378‐3p (high levels)	Regulate macrophages apoptosis	^158^
miR‐124, miR‐324‐5p, miR‐511‐3p	Macrophages function regulation	^158^

Interestingly, type 2 cytokines promote tumour metastasis and contribute to chemoresistance, and miR‐126 has been shown to promote tumour angiogenesis via a TH2‐dependent IL‐13 release mechanism, in a model of breast tumour metastasis.^174^ Furthermore, high miR‐126 expression in acute myeloid leukaemia patients has been associated with a higher incidence of relapse and, additionally, poor survival.^175^ Circulating miRNAs have been examined in numerous cancers, type II diabetes, neurodegenerative diseases, lung diseases, such as asthma and cardiovascular diseases.^167^ ^–^ ^175^

**FIGURE 3:**
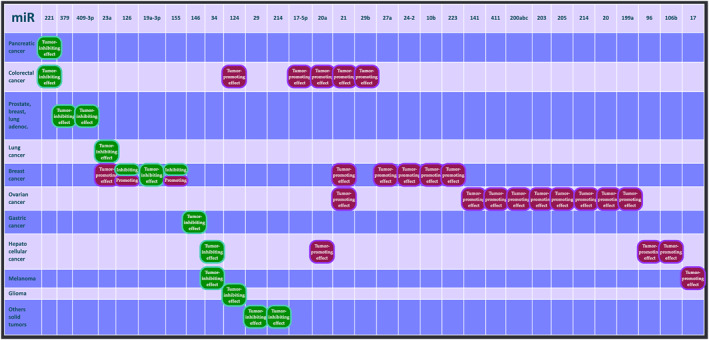
Effects of microRNA in several cancers

The relation between miRNA and cancer are shown in Table 5 and summarized in Figure 3.

**TABLE 5:** MiRNA and cancer

Type of cancer	MiRNA expression		Reference
	Tumour‐promoting effect	Tumour‐inhibiting effect	
Pancreatic cancer		↑miR‐221	^170^
Colorectal cancer	↑miR‐17‐5p, miR‐20a, miR‐124, miR‐21, miR‐29b	↑miR‐221 (greater survival)	^170^
Prostate, breast, lung adenocarcinoma		↑miR‐379↑ miR‐409‐3p	^171^
Lung cancer		↑miR‐23a	^174^
Breast cancer	↑miR‐23a/‐27a/‐24‐2 cluster	↑miR‐126 (antitumour activity, inhibition of invasion and metastasis)	^176^
	↑miR‐10b, miR‐21, miR‐155, miR‐223	↑miR‐19a‐3p	
	↓miR‐126, miR‐146	↑miR‐155 (metastasis inhibition)	
Ovarian cancer	↑miR‐21, ‐141, ‐411, ‐200a, ‐200b, ‐200c, ‐203, ‐205, ‐214		^172^
	↑ miR‐20 ↓miR‐199^a^		
Gastric cancer		↓miR‐146	^173^
Hepatocellular cancer	↑miR‐20a, miR‐96, miR‐106b	↓miR‐34	^173^
Melanoma	↓miR‐17	↓miR‐34	^173^
Glioma		↓miR‐124	^173^
Others solid tumours		↑ miR‐29 and mir‐214	^173^

Several studies have suggested a crosstalk between immune cells and cancer via miRNAs. As changes to miRNA expression are seen in numerous diseases from cancers to respiratory diseases, it is becoming more apparent that they might be used as viable biomarkers for new therapeutic treatments. Mimics of let‐7 and miR‐34 have been tried in murine models of lung cancer, leading to reduction in the volume of the tumours.^176^

## 5 CONCLUSIONS AND FUTURE PERSPECTIVES

The ‘‘immunebalance’*’* is a delicate mechanism that immune system uses to maintain homeostasis. Immune tolerance in allergy and cancer could represent a valid key to develop new anti‐cancer therapies. While allergen immunotherapy (AIT) may re‐establish tolerance involving Tregs, IL‐10 and TGFβ, and class switching to anti‐inflammatory IgG4 and IgA; in cancer, immune suppression and development of constant immunoregulatory response favour cancer progression. Checkpoint inhibitors are competent to break immune tolerance and they have demonstrated efficacies in some cancer types. Anti‐CTLA4 (anti‐cytotoxic T‐lymphocytes‐associated protein 4) ipilimumab and anti‐PD1 (anti‐programmed cell death protein 1) nivolumab and pembrolizumab are approved for the treatment of advanced melanoma. Nivolumab is approved for metastatic (NSCLS, renal cell carcinoma and classical Hodgkin lymphoma; pembrolizumab for NSCLS, head and neck squamous cell carcinoma and classical Hodgkin lymphoma. Anti‐PD1 ligand antibody avelumab is applied for the treatment of NSCLC and other check‐point inhibitors, atezolizumab and durvalumab are under investigation.^177^, ^178^, ^179^

Another recent anti‐cancer therapy is provided by CAR‐T‐cell therapy which consists in the use of T cells engineered to express chimeric antigen receptors (CARs) with tumour specificity.

The strong antitumour effect of IgE antibodies consented to generate engineering antibodies, and a recombinant anti‐cancer IgE has shown encouraging results in the clinical setting.^84^, ^85^, ^86^

Other interesting anti‐cancer strategies to be developed possibly in the future: re‐education of macrophages favouring M1 phenotype; stimulation of DCs activated phenotype; potentiation of NK cells action with transfer of allogenic NK cells genetically modified NK cells and antibody therapies; promotion of T and B cell activity, targeting IL‐4 and IL‐13 contrasting T and Breg cells activity; enhancing of the T_FH_ cells; targeting mast cells and their angiogenic factors. In future, eosinophils can be activated by immunotherapy such as checkpoint inhibitors or GM‐CSF‐based vaccines, or by adoptive transfer of these cells in an appropriate setting. It has been demonstrated that combined immunotherapy using checkpoint blockade in the presence of CD26i inhibits tumour growth. Therefore, contrasting HMGB1 actions, RAGE signalling and others alarmin might be a potential target for the treatment of cancer.

## CONFLICT OF INTEREST

The authors declare no conflict of interest.

## AUTHOR CONTRIBUTIONS

**Mario Di Gioacchino:** Conceptualization; Supervision. **Loredana Della Valle:** Writing – original draft; Writing – review & editing. **Alessandro Allegra:** Writing – original draft; Writing – review & editing. **Giovanni Pioggia:** Data curation; Methodology. **Sebastiano Gangemi:** Conceptualization; Supervision.

## References

[1] Della Valle L , Gatta A , Farinelli A , et al. Allergooncology: an expanding research area. J Biol Regul Homeost Agents. 2020;34(2):319–326. 10.23812/19-418-63-E 32431140

[2] Jensen‐Jarolim E , Bax HJ , Bianchini R , et al. AllergoOncology – the impact of allergy in oncology: EAACI position paper. Allergy. 2017;72(6):866–887. 10.1111/all.13119 28032353 PMC5498751

[3] Jensen‐Jarolim E , Bax HJ , Bianchini R , et al. AllergoOncology: opposite outcomes of immune tolerance in allergy and cancer. Allergy. 2018;73:328–340. 10.1111/all.13311 28921585 PMC6038916

[4] Lin S , Gregory RI . MicroRNA biogenesis pathways in cancer. Nat Rev Cancer. 2015;15(6):321–333. 25998712 10.1038/nrc3932 PMC4859809

[5] Lindelçf B , Granath F , Tengvall‐Linder M , Ekbom A . Allergy and cancer. Allergy. 2005;60:1116–1120. 16076294 10.1111/j.1398-9995.2005.00808.x

[6] Gergen PJ , Turkeltaub PC , Sempos CT . Is allergy skin test reactivity a predictor of mortality? Findings from a national cohort. Clin Exp Allergy. 2000;30:1717–1723. 11122209 10.1046/j.1365-2222.2000.00971.x

[7] Jacobs EJ , Gapstur SM , Newton CC , Turner MC , Campbell PT . Hay Fever and asthma as markers of atopic immune response and risk of colorectal cancer in three large cohort studies. Cancer Epidemiol Biomark Prev. 2013;22:661–669. 10.1158/1055-9965.EPI-12-1229 23513040

[8] Linabery AM , Prizment AE , Anderson KE , Cerhan JR , Poynter JN , Ross JA . Allergic diseases and risk of hematopoietic malignancies in a cohort of postmenopausal women: a report from the Iowa Women’s Health Study. Cancer Epidemiol Biomark Prev. 2014;23:1903–1912. 10.1158/1055-9965.EPI-14-0423 PMC4154995 24962839

[9] Platz EA , Drake CG , Wilson KM , et al. Asthma and risk of lethal prostate cancer in the health professionals follow‐up study. Int J Cancer. 2015;137:949–958. 25648070 10.1002/ijc.29463 PMC4478199

[10] Prizment AE , Anderson KE , Visvanathan K , Folsom AR . Inverse association of eosinophil count with colorectal cancer incidence: atherosclerosis risk in communities study. Cancer Epidemiol Biomark Prev. 2011;20:1861–1864. 10.1158/1055-9965.EPI-11-0360 PMC3175810 21742945

[11] Wulaningsih W , Holmberg L , Garmo H , et al. Investigating the association between allergen‐specific immunoglobulin E, cancer risk and survival. OncoImmunology. 2016;5(7):e1154250. 27471625 10.1080/2162402X.2016.1154250 PMC4938379

[12] Musolino C , Allegra A , Minciullo PL , Gangemi S . Allergy and risk of hematologic malignancies: associations and mechanisms. Leuk Res. 2014;38(10):1137–1144. 25171954 10.1016/j.leukres.2014.08.004

[13] Shadman M , White E , De Roos AJ , Walte RB . Associations between allergies and risk of hematologic malignancies: results from the VITamins and life style cohort study. Am J Hematol. 2013;88:1050–1054. 23918679 10.1002/ajh.23564 PMC4001851

[14] Kristinsson SY , Koshiol J , Bjorkholm M , et al. Immune‐related and inflammatory conditions and risk of lymphoplasmacytic lymphoma or Waldenstrom macroglobulinemia. J Natl Cancer Inst. 2010;102:557–567. 20181958 10.1093/jnci/djq043 PMC2857799

[15] Mejıa‐Arangure JM , Fajardo‐Gutierrez A , Flores‐Aguilar H , et al. Environmental factors contributing to the development of childhood leukemia in children with Down’s syndrome. Leukemia. 2003;17:1905–1907. 12970794 10.1038/sj.leu.2403047

[16] Mejia‐Arangure JM , Fajardo‐Gutierrez A , Perez‐Saldivar ML , et al. Magnetic fields and acute leukemia in children with Down syndrome. Epidemiology. 2007;18:158–161. 17099322 10.1097/01.ede.0000248186.31452.be

[17] Koshiol J , Lam TK , Gridley G , Check D , Brown LM , Landgren OR . Racial differences in chronic immune stimulatory conditions and risk of non‐Hodgkin’s lymphoma in veterans from the United States. J Clin Oncol. 2011;29:378–385. 21172877 10.1200/JCO.2010.30.1515 PMC3058284

[18] Gutensohn N , Cole P . Childhood social environment and Hodgkin’s disease. N Engl J Med. 1981;304:135–140. 6255329 10.1056/NEJM198101153040302

[19] Hjalgrim H , Smedby KE , Rostgaard K , et al. Infectious mononucleosis, child‐hood social environment, and risk of Hodgkin lymphoma. Cancer Res. 2007;67:2382–2388. 17332371 10.1158/0008-5472.CAN-06-3566

[20] Vonderheid EC , Hamilton RG , Kadin ME . Mycosis fungoides and its relationship to atopy, serum total IgE, and eosinophil counts. Clin Lymphoma Myeloma Leuk. 2020;12(20):S2152–S2650. 10.1016/j.clml.2020.11.007 33342729

[21] Soderberg KC , Jonsson F , Winqvist O , Hagmar L , Feychting M . Autoimmune diseases, asthma and risk of haematological malignancies: a nationwide case‐control study in Sweden. Eur J Cancer. 2006;42:3028–3033. 16945522 10.1016/j.ejca.2006.04.021

[22] Mirabelli MC , Zock JP , D’Errico A , et al. Occupational exposure to high molecular weight allergens and lymphoma risk among Italian adults. Cancer Epidemiol Biomark Prev. 2009;18:2650–2654. 10.1158/1055-9965.EPI-09-0446 PMC2762781 19755650

[23] Becker N , de Sanjose S , Nieters A , et al. Birth order, allergies and lymphoma risk: results of the European collaborative research project Epilymph. Leuk Res. 2007;31:1365–1372. 17481729 10.1016/j.leukres.2007.02.019

[24] Ellison‐Loschmann L , Benavente Y , Douwes J , et al. Immunoglobulin E levels and risk of lymphoma in a case‐control study in Spain. Cancer Epidemiol Biomark Prev. 2007;16:1492–1864. 10.1158/1055-9965.EPI-07-0176 17627016

[25] Melbye M , Smedby KE , Lehtinen T , et al. Atopy and risk of non‐Hodgkin lymphoma. J Natl Cancer Inst. 2007;99:158–166. 17227999 10.1093/jnci/djk019

[26] Magnani C , Pastore G , Luzzatto L , Terracini B . Parental occupation and other environmental factors in the etiology of leukemias and non‐Hodgkin’s lymphomas in childhood: a case–control study. Tumori. 1990;76:413–419. 2256184 10.1177/030089169007600501

[27] Rudant J , Orsi L , Monnereau A , et al. Childhood Hodgkin’s lymphoma, non‐Hodgkin’s lymphoma and factors related to the immune system: the Escale Study (SFCE). Int J Cancer. 2011;129:2236–2247. 21170962 10.1002/ijc.25862

[28] Linabery AM , Jurek AM , Duval S , Ross JA . The association between atopy and childhood/adolescent leukemia: a meta‐analysis. Am J Epidemiol. 2010;171:749–764. 20228139 10.1093/aje/kwq004 PMC2877483

[29] Rudant J , Orsi L , Menegaux F , et al. Childhood acute leukemia, early common infections, and allergy: the ESCALE Study. Am J Epidemiol. 2010;172:1015–1027. 20807738 10.1093/aje/kwq233

[30] Brown LM , Gridley G , Check D , Landgren O . Risk of multiple myeloma and mono‐clonal gammopathy of undetermined significance among white and black male United States veterans with prior autoimmune, infectious, inflammatory, and allergic disorders. Blood. 2008;111:3388–3394. 18239085 10.1182/blood-2007-10-121285 PMC2275008

[31] Strachan DP . Hay fever, hygiene, and household size. BMJ. 1989;299(6710):1259–1260. 2513902 10.1136/bmj.299.6710.1259 PMC1838109

[32] Romagnani S . The increased prevalence of allergy and the hygiene hypothesis: missing immune deviation, reduced immune suppression, or both? Immunology. 2004;112(3):352–363. 10.1111/j.1365-2567.2004.01925.x 15196202 PMC1782506

[33] Soderberg KC , Hagmar L , Schwartzbaum J , Feychting M . Allergic conditions and risk of hematological malignancies in adults: a cohort study. BMC Publ Health. 2004;4:51. 10.1186/1471-2458-4-51 PMC534807 15527506

[34] Geissmann MG , Manz S , Jung MH , Sieweke M , Merad K . Ley development of monocytes, macrophages, and dendritic cells. Science. 2010;327:656–661. 20133564 10.1126/science.1178331 PMC2887389

[35] Sugiura H , Ichinose M . Oxidative and nitrative stress in bronchial asthma. Antioxid Redox Signal. 2008;10(4):785–797. 18177234 10.1089/ars.2007.1937

[36] Sica A , Mantovani A . Macrophage plasticity and polarization: in vivo veritas. J Clin Invest. 2012;122(3):787–795. 22378047 10.1172/JCI59643 PMC3287223

[37] Varricchi G , Loffredo S , Galdiero MS , et al. Innate effector cells in angiogenesis and lymphangiogenesis. Curr Opin Immunol. 2018;53:152–160. 29778674 10.1016/j.coi.2018.05.002

[38] Veremeyko T , Siddiqui S , Sotnikov I , Yung A , Ponomarev ED . IL‐4/IL‐13‐dependent and independent expression of miR‐124 and its contribution to M2 phenotype of monocytic cells in normal conditions and during allergic inflammation. PLoS One. 2013;8(12):e81774. 24358127 10.1371/journal.pone.0081774 PMC3864800

[39] Bianchini R , Roth‐Walter F , Ohradanova‐Repic A , et al. IgG4 drives M2a macrophages to a regulatory M2b‐like phenotype: potential implication in immune tolerance. Allergy. 2019;74(3):483–494. 30338531 10.1111/all.13635 PMC6492166

[40] Jordakieva G , Bianchini R , Reichhold D , et al. IgG4 induces tolerogenic M2‐like macrophages and correlates with disease progression in colon cancer. OncoImmunology. 2021;10(1):1880687. 10.1080/2162402X.2021.1880687 33628623 PMC7889146

[41] Martinez FO , Gordon S . The M1 and M2 paradigm of macrophage activation: time for reassessment. F1000 Prime Rep. 2014;6:13. 10.12703/P6-13 PMC3944738 24669294

[42] Mantovani A , Locati M . Tumor‐associated macrophages as a paradigm of macrophage plasticity, diversity, and polarization: lessons and open questions. Arterioscler Thromb Vasc Biol. 2013;33(7). 10.1161/ATVBAHA.113.300168 23766387

[43] Beyer M , Mallmann MR , Xue J . High‐resolution transcriptome of human macrophages. PLoS One. 2012;7:e45466. 23029029 10.1371/journal.pone.0045466 PMC3448669

[44] Josephs DH , Bax HJ , Dodev T , et al. Anti‐folate receptor‐alpha IgE but not IgG recruits macrophages to attack tumors via TNFalpha/MCP‐1 signaling. Cancer Res. 2017;77:1127–1141. 28096174 10.1158/0008-5472.CAN-16-1829 PMC6173310

[45] Mills CD , Lenz LL , Harris RA . A breakthrough: macrophage‐directed cancer immunotherapy. Cancer Res. 2016;76:513–516. 26772756 10.1158/0008-5472.CAN-15-1737 PMC4738030

[46] Pan C , Wang Y , Liu Q . Phenotypic profiling and prognostic significance of immune infiltrates in esophageal squamous cell carcinoma. OncoImmunology. 2021;10(1). 10.1080/2162402X.2021.1883890 PMC7889084 33628625

[47] Deniz G , van de Veen W , Akdis M . Natural killer cells in patients with allergic diseases. J Allergy Clin Immunol. 2013;132(3):527–535. 10.1016/j.jaci.2013.07.030 23993354

[48] Deniz G , Akdis M . NK cell subsets and their role in allergy. Expert Opin Biol Ther. 2011;11(7):833–841. 10.1517/14712598.2011.572549 21426239

[49] Ghaemdoust F , Keshavarz‐Fathi M , Rezaei N . Natural killer cells and cancer therapy, what we know and where we are going. Immunotherapy. 2019;11(14):1231–1251. 31422725 10.2217/imt-2019-0040

[50] Bottcher JP , Bonavita E , Chakravarty P , et al. NK cells stimulate recruitment of cDC1 into the tumor microenvironment promoting cancer immune control. Cell. 2018;172:1022–1037. 29429633 10.1016/j.cell.2018.01.004 PMC5847168

[51] Ealey KN , Koyasu S . How many subsets of innate lymphoid cells do we need? Immunity. 2017;46:1. 28099859 10.1016/j.immuni.2016.12.018

[52] Nussbaum K , Burkhard SH , Ohs I , et al. Tissue microenvironment dictates the fate and tumor‐suppressive function of type 3 ILCs. J Exp Med. 2017;214(8):2331–2347. 10.1084/jem.20162031 28698286 PMC5551572

[53] Munn DH . Blocking Ido activity to enhance anti‐tumor immunity. Front Biosci (Elite Ed). 2012;4:734–745. 10.2741/414 22201909

[54] Yue W , Lin Y , Yang X , Li B , Liu J , He R . Thymic stromal lymphopoietin (TSLP) inhibits human colon tumor growth by promoting apoptosis of tumor cells. Oncotarget. 2016;7:16840–16854. 26919238 10.18632/oncotarget.7614 PMC4941354

[55] Boonpiyathad T , Satitsuksanoa P , Akdis M , Akdis CA . Il‐10 producing T and B cells in allergy. Semin Immunol. 2019;44:101326. 31711770 10.1016/j.smim.2019.101326

[56] Inoue S , Leitner WW , Golding B , Scott D . Inhibitory effects of B cells on antitumor immunity. Cancer Res. 2006;66(15):7741–7767. 16885377 10.1158/0008-5472.CAN-05-3766

[57] Carrier Y , Yuan J , Kuchroo VK , Weiner HL . Th3 cells in peripheral tolerance. I. Induction of Foxp3‐positive regulatory T cells by Th3 cells derived from TGF‐beta T cell‐transgenic mice. J Immunol. 2007;178(1):179–185. 17182553 10.4049/jimmunol.178.1.179

[58] Yadav M , Stephan S , Bluestone JA . Peripherally induced Tregs—role in immune homeostasis and autoimmunity. Front Immunol. 2013;4:232. 10.3389/fimmu.2013.00232 23966994 PMC3736167

[59] Sakaguchi S , Yamaguchi T , Nomura T , Ono M . Regulatory T cells and immune tolerance. Cell. 2008;133:775–787. 18510923 10.1016/j.cell.2008.05.009

[60] Katz SI , Parker D , Turk JL . B‐cell suppression of delayed hypersensitivity reactions. Nature. 1974;251:550–551. 4547522 10.1038/251550a0

[61] Linnebacher M , Maletzki C . Tumor‐infiltrating B cells: the ignored players in tumor immunology. OncoImmunology. 2012;1:1186–1188. 23170274 10.4161/onci.20641 PMC3494640

[62] Gowthaman U , Chen SJ , Zhang B , et al. Identification of a T follicular helper cell subset that drives anaphylactic IgE. Science. 2019;365. 10.1126/science.aaw6433 PMC6901029 31371561

[63] Guo Z , Liang H , Xu Y , et al. The role of circulating T follicular helper cells and regulatory cells in non‐small cell lung cancer patients. Scand J Immunol. 2017;86:107–112. 28513867 10.1111/sji.12566

[64] Vyas AK , Khosla N , Trehanpati KN . Response to ‘The role of circulating T follicular helper cells and regulatory cells in non‐small cell lung cancer patients”. Immunology 2017;8:248. 10.1111/sji.12584 28810080

[65] Gu‐Trantien C , Loi S , Garaud S , et al. CD4⁺ follicular helper T cell infiltration predicts breast cancer survival. J Clin Invest. 2013;123(7):2873–2892. 23778140 10.1172/JCI67428 PMC3696556

[66] Zhang H , Yue R , Zhao P , et al. Proinflammatory follicular helper T cells promote immunoglobulin G secretion, suppress regulatory B cell development, and correlate with worse clinical outcomes in gastric cancer. Tumour Biol 2017;39(6):1010428317705747. 28631561 10.1177/1010428317705747

[67] Li L , Ma Y , Xu Y , Maerkeya K . TIM‐3 expression identifies a distinctive PD‐1+ follicular helper T cell subset, with reduced interleukin 21 production and B cell help function in ovarian cancer patients. Int Immunopharmacol. 57;2018:139–146. 29482158 10.1016/j.intimp.2018.02.016

[68] Ma QY , Huang DY , Zhang HJ , Chen J , Miller W , Chen XF . Function of follicular helper T cell is impaired and correlates with survival time in non‐small cell lung cancer. Int Immunopharmacol. 2016;41:1–7. 27788370 10.1016/j.intimp.2016.10.014

[69] Cogswell DT , Gapin L , Tobin HM , McCarter MD , Tobin RP . MAIT cells: partners or enemies in cancer immunotherapy? Cancers. 2021;13(7):1502. 10.3390/cancers13071502 33805904 PMC8037823

[70] Ye L , Pan J , Pasha MA , et al. Mucosal‐associated invariant T cells restrict allergic airway inflammation. J Allergy Clin Immunol. 2020;145(5):1469‐1473. 10.1016/j.jaci.2019.12.891 31874183 PMC7214121

[71] Conti P , Caraffa A , Kritas SK , et al. Mast cell, pro‐inflammatory and anti‐inflammatory: Jekyll and Hyde, the story continues. J Biol Regul Homeost Agents. 2017;31(2):263–267. 28685525

[72] Detoraki A , Staiano R , Granata F , et al. Vascular endothelial growth factors synthesized by human mast cells exert angiogenic effects. J Allergy Clin Immunol. 2009;944(123):1142. 10.1016/j.jaci.2009.01.044 19275959

[73] Varricchi G , Galdiero MR , Loffredo S , et al. Are mast cells MASTers in cancer? Front Immunol. 2017;8:424. 28446910 10.3389/fimmu.2017.00424 PMC5388770

[74] Varricchi G , de Paulis A , Marone G , Galli SJ . Future needs in mast cells. Int J Mol Sci. 2019;20:4397. 10.3390/ijms20184397 PMC6769913 31500217

[75] O’Sullivan JA , Bochner BS . Eosinophils and eosinophil‐associated diseases: an update. J Allergy Clin Immunol. 2018;141(2):505–517. 29045815 10.1016/j.jaci.2017.09.022 PMC5803328

[76] Teo PZ , Utz PJ , Mollick JA . Using the allergic immune system to target cancer: activity of IgE antibodies specific for human CD20 and MUC1. Cancer Immunol Immunother. 2012;61:2295–2309. 22692757 10.1007/s00262-012-1299-0 PMC3833261

[77] Abdala Valencia H , Loffredo LF , Misharin AV , Berdnikovs S . Phenotypic plasticity and targeting of Siglec‐F(high) CD11c(low) eosinophils to the airway in a murine model of asthma. Allergy. 2016;71(2):267–271. 26414117 10.1111/all.12776

[78] Afferni C , Buccione C , Andreone S , et al. The pleiotropic immunomodulatory functions of IL‐33 and its implications in tumor immunity. Front Immunol. 2018;9:2601. 30483263 10.3389/fimmu.2018.02601 PMC6242976

[79] Carretero R , Sektioglu IM , Garbi N , Salgado OC , Beckhove P , Hämmerling GJ . Eosinophils orchestrate cancer rejection by normalizing tumor vessels and enhancing infiltration of CD8(+) T cells. Nat Immunol. 2015;16(6):609–617. 25915731 10.1038/ni.3159

[80] Varricchi G , Galdiero MR , Loffredo S , et al. Eosinophils: the unsung heroes in cancer? OncoImmunology. 2018;7:e1393134. 29308325 10.1080/2162402X.2017.1393134 PMC5749653

[81] Lucarini V , Ziccheddu G , Macchia I , et al. IL‐33 restricts tumor growth and inhibits pulmonary metastasis in melanoma‐bearing mice through eosinophils. Oncoimmunology. 2017. 10.1080/2162402X.2017.1317420 PMC5486175 28680750

[82] Jiang L , Shen Y , Guo D , et al. EpCAM‐dependent extracellular vesicles from intestinal epithelial cells maintain intestinal tract immune balance. Nat Commun. 2016;7:13045. 27721471 10.1038/ncomms13045 PMC5062543

[83] Jensen‐Jarolim E , Achatz G , Turner MC , et al. AllergoOncology: the role of IgE‐mediated allergy in cancer. Allergy. 2008;63(10):1255–1266. 18671772 10.1111/j.1398-9995.2008.01768.x PMC2999743

[84] Nakamura M , Souri EA , Osborn G , et al. IgE activates monocytes from cancer patients to acquire a pro‐inflammatory phenotype. Cancers. 2020;12(11):3376. 10.3390/cancers12113376 PMC7698027 33203088

[85] Teo PZ , Utz PJ , Mollick JA . Using the allergic immune system to target cancer: activity of IgE antibodies specific for human CD20 and MUC1. Cancer Immunol Immunother. 2012;61:2295–2309. 22692757 10.1007/s00262-012-1299-0 PMC3833261

[86] Linabery AM , Prizment AE , Anderson KE , Cerha JR , Poynter JN , Ross JA . Allergic diseases and risk of hematopoietic malignancies in a cohort of postmenopausal women: a report from the Iowa Women’s Health Study. Cancer Epidemiol Biomark Prev. 2014;23(9):1903–1912. 10.1158/1055-9965.EPI-14-0423 PMC4154995 24962839

[87] Cui Y , Hill AW . Atopy and specific cancer sites: a review of epidemiological studies. Clin Rev Allergy Immunol. 2016;51(3):981–982. 10.1007/s12016-016-8559-2 27277132

[88] Ferastraoaru D , Bax HJ , Bergmann C , et al. AllergoOncology: ultra‐low IgE, a potential novel biomarker in cancer‐a position paper of the European Academy of allergy and Clinical Immunology (EAACI). Clin Transl Allergy. 2020;10:32. 32695309 10.1186/s13601-020-00335-w PMC7366896

[89] Jensen‐Jarolim E , Turner MC , Karagiannis SN . AllergoOncology: IgE‐ and IgG4‐mediated immune mechanisms linking allergy with cancer and their translational implications. J Allergy Clin Immunol. 2017;140(4):982–984. 28526623 10.1016/j.jaci.2017.04.034

[90] Saul L , Ilieva K , Bax H , et al. IgG subclass switching and clonal expansion in cutaneous melanoma and normal skin. Sci Rep. 2016;6:29736. 27411958 10.1038/srep29736 PMC4944184

[91] Nigro EA , Brini AT , Soprana E , et al. Antitumor IgE adjuvanticity: key role of Fc epsilon RI. J Immunol. 2009;183:4530–4536. 19748979 10.4049/jimmunol.0900842

[92] Powe DG , Groot Kormelink T , Sisson M , et al. Evidence for the involvement of free light chain immunoglobulins in allergic and nonallergic rhinitis. J Allergy Clin Immunol. 2010;125(1):139‐145. 19818484 10.1016/j.jaci.2009.07.025

[93] Knittelfelder R , Riemer AB , Jensen‐Jarolim E . Mimotope vaccination–from allergy to cancer. Expert Opin Biol Ther. 2009;9:493–506. 19344285 10.1517/14712590902870386 PMC3049225

[94] Joosten LAB , Netea MG . Intracellular alarmins: hidden dangers signals crucial for cancer, host defense and inflammatory processes. Semin Immunol. 2018;38:1–2. 30554607 10.1016/j.smim.2018.10.007

[95] Roth‐Walter F , Schmutz R , Mothes‐Luksch N , et al. Clinical efficacy of sublingual immunotherapy is associated with restoration of steady‐state serum lipocalin 2 after SLIT: a pilot study. World Allergy Organ J. 2018;11(1):21. 30323863 10.1186/s40413-018-0201-8 PMC6166283

[96] Chen GY , Nun˜ez G . Sterile inflammation: sensing and reacting to damage. Nat Rev Immunol. 2010;10:826–837. 21088683 10.1038/nri2873 PMC3114424

[97] Goodwin GH , Sanders C , Johns EW . A new group of chromatin‐associated proteins with a high content of acidic and basic amino acids. Eur J Biochem. 1973;38:14–19. 4774120 10.1111/j.1432-1033.1973.tb03026.x

[98] Wang S , Zhang Y . HMGB1 in inflammation and cancer. J Hematol Oncol. 2020;13(1):116. 32831115 10.1186/s13045-020-00950-x PMC7443612

[99] Lu B , Antoine DJ , Kwan K , et al. JAK/STAT1 signaling promotes HMGB1 hyperacetylation and nuclear translocation. Proc Natl Acad Sci U S A. 2014;111:3068–3073. 24469805 10.1073/pnas.1316925111 PMC3939889

[100] Tang D , Kang R , Zeh HJ , Lotze MT . High‐mobility group box 1, oxidative stress, and disease. Antioxid Redox Signal. 2011;14:1315–1335. 20969478 10.1089/ars.2010.3356 PMC3048826

[101] Lotze MT , Tracey KJ . High‐mobility group box 1 protein (HMGB1): nuclear weapon in the immune arsenal. Nat Rev Immunol. 2005;5:331–342. 15803152 10.1038/nri1594

[102] Klune JR , Dhupar R , Cardinal J , Billiar TR , Tsung A . HMGB1: endogenous danger signaling. Mol Med. 2008;14:476–484. 18431461 10.2119/2008-00034.Klune PMC2323334

[103] Dumitriu IE , Bianchi ME , Bacci M , Manfredi AA , Rovere‐Querini P . The secretion of HMGB1 is required for the migration of maturing dendritic cells. J Leukoc Biol. 2007;81:84–91. 17035340 10.1189/jlb.0306171

[104] Dumitriu IE , Baruah P , Valentinis B , et al. Release of high mobility group box 1 by dendritic cells controls T cell activation via the receptor for advanced glycation end products. J Immunol. 2005;174:7506–7510. 15944249 10.4049/jimmunol.174.12.7506

[105] Manfredi AA , Capobianco A , Esposito A , et al. Maturing dendritic cells depend on RAGE for in vivo homing to lymph nodes. J Immunol. 2008;180:2270–2275. 18250435 10.4049/jimmunol.180.4.2270

[106] Treutiger CJ , Mullins GE , Johansson A‐SM , et al. High mobility group 1 B‐box mediates activation of human endothelium. J Intern Med. 2003;254:375–385. 12974876 10.1046/j.1365-2796.2003.01204.x

[107] Fiuza C , Bustin M , Talwar S , et al. Inflammation‐promoting activity of HMGB1 on human microvascular endothelial cells. Blood. 2003;101:2652–2660. 12456506 10.1182/blood-2002-05-1300

[108] Chiba S , Baghdadi M , Akiba H , et al. Tumor‐infiltrating DCs suppress nucleic acid mediated innate immune responses through interactions between the receptor TIM‐3 and the alarmin HMGB1. Nat Immunol. 2012;13:832–842. 22842346 10.1038/ni.2376 PMC3622453

[109] Ma L , Zeng J , Mo B , et al. High mobility group box 1: a novel mediator of Th2‐type response‐induced airway inflammation of acute allergic asthma. J Thorac Dis. 2015;7(10):1732–1741. 26623095 10.3978/j.issn.2072-1439.2015.10.18 PMC4635276

[110] Kang R , Zhang Q , Zeh HJ , Lotze MT , Tang D . HMGB1 in cancer: good, bad, or both? Clin Cancer Res. 2013;19:4046–4057. 23723299 10.1158/1078-0432.CCR-13-0495 PMC3732559

[111] Ellerman JE , Brown CK , de Vera M , et al. Masquerader: high mobility group box‐1 and cancer. Clin Cancer Res. 2007;13:2836–2848. 17504981 10.1158/1078-0432.CCR-06-1953

[112] Jube S , Rivera ZS , Bianchi ME , et al. Cancer cell secretion of the DAMP protein HMGB1 supports progression in malignant mesothelioma. Cancer Res. 2012;72:3290–3301. 22552293 10.1158/0008-5472.CAN-11-3481 PMC3389268

[113] Taguchi A , Blood DC , del Toro G , et al. Blockade of RAGE‐amphoterin signalling suppresses tumour growth and metastases. Nature. 2000;405:354–360. 10830965 10.1038/35012626

[114] Apetoh L , Ghiringhelli F , Tesniere A , et al. The interaction between HMGB1 and TLR4 dictates the outcome of anticancer chemotherapy and radiotherapy. Immunol Rev. 2007;220:47–59. 17979839 10.1111/j.1600-065X.2007.00573.x

[115] Rider P , Carmi Y , Voronov E , Apte RN . Interleukin‐1α. Semin Immunol. 2013;25:430–438. 24183701 10.1016/j.smim.2013.10.005

[116] Bertheloot D , Latz E . HMGB1, IL‐1α, IL‐33 and S100 proteins: dual‐function alarmins. Cell Mol Immunol. 2017;14(1):43–64. 27569562 10.1038/cmi.2016.34 PMC5214941

[117] Afonina IS , Tynan GA , Logue SE , et al. Granzyme B‐dependent proteolysis acts as a switch to enhance the proinflammatory activity of IL‐1α. Mol Cell. 2011;44:265–278. 22017873 10.1016/j.molcel.2011.07.037 PMC3319689

[118] Carmi Y , Voronov E , Dotan S , et al. The role of macrophage‐derived IL‐1 in induction and maintenance of angiogenesis. J Immunol. 2009;183:4705–4714. 19752225 10.4049/jimmunol.0901511

[119] Hong DS , Hui D , Bruera E , et al. MABp1, a first‐in‐class true human antibody targeting interleukin‐1α in refractory cancers: an open‐label, phase 1 dose‐escalation and expansion study. Lancet Oncol. 2014;15:656–666. 24746841 10.1016/S1470-2045(14)70155-X

[120] Pollock AS , Turck J , Lovett DH . The prodomain of interleukin 1alpha interacts with elements of the RNA processing apparatus and induces apoptosis in malignant cells. FASEB J. 2003;17:203–213. 12554699 10.1096/fj.02-0602com

[121] Hammad H . Epithelial cell regulation of immune responses in the lung. In: Jiri M , Warren S , Michael WR , Brian LK , Hilde C , Bart NL , eds. Mucosal Immunology. 4th ed. Academic Press; 2015:591–603.

[122] Gross SR , Sin CGT , Barraclough R , Rudland PS . Joining S100 proteins and migration: for better or for worse, in sickness and in health. Cell Mol Life Sci. 2014;71:1551–1579. 23811936 10.1007/s00018-013-1400-7 PMC11113901

[123] Donato R . Intracellular and extracellular roles of S100 proteins. Microsc Res Tech. 2003;60:540–551. 12645002 10.1002/jemt.10296

[124] Leclerc E , Fritz G , Vetter SW , Heizmann CW . Binding of S100 proteins to RAGE: an update. Biochim Biophys Acta. 2009;1793:993–1116. 19121341 10.1016/j.bbamcr.2008.11.016

[125] Dassan P , Keir G , Brown MM . Criteria for a clinically informative serum biomarker in acute ischaemic stroke: a review of S100B. Cerebrovasc Dis. 2009;27:295–302. 19202335 10.1159/000199468

[126] Moore BW . A soluble protein characteristic of the nervous system. Biochem Biophys Res Commun. 1965;19:739–744. 4953930 10.1016/0006-291x(65)90320-7

[127] Kim SO , Merchant K , Nudelman R , et al. OxyR: a molecular code for redox‐related signaling. Cell. 2002;109(3):383–396. 12015987 10.1016/s0092-8674(02)00723-7

[128] Ma W , Lee SE , Guo J , et al. RAGE ligand upregulation of VEGF secretion in ARPE‐19 cells. Invest Ophthalmol Vis Sci. 2007;48:1355–1361. 17325184 10.1167/iovs.06-0738

[129] Hammad H , Lambrecht BN . Barrier epithelial cells and the control of type 2 immunity. Immunity. 2015;43(1):29–40. 26200011 10.1016/j.immuni.2015.07.007

[130] Bresnick AR , Weber DJ , Zimmer DB . S100 proteins in cancer. Nat Rev Cancer. 2015;15:96–109. 25614008 10.1038/nrc3893 PMC4369764

[131] Nasser MW , Elbaz M , Ahirwar DK , Ganju RK . Conditioning solid tumor microenvironment through inflammatory chemokines and S100 family proteins. Cancer Lett 2015;365:11–22. 25963887 10.1016/j.canlet.2015.05.002 PMC11707611

[132] Leclerc E , Vetter SW . The role of S100 proteins and their receptor RAGE in pancreatic cancer. Biochim Biophys Acta. 2015;1852:2706–2711. 26435083 10.1016/j.bbadis.2015.09.022 PMC4643662

[133] Bresnick AR , Weber DJ , Zimmer DB . S100 proteins in cancer. Nat Rev Cancer. 2015;15(2):96–109. 25614008 10.1038/nrc3893 PMC4369764

[134] Allgöwer C , Kretz AL , von Karstedt S , Wittau M , Henne‐Bruns D , Lemke J . Friend or foe: S100 proteins in cancer. Cancers. 2020;12(8):2037. 10.3390/cancers12082037 PMC7465620 32722137

[135] Afferni C , Buccione C , Andreone S , et al. The pleiotropic immunomodulatory functions of IL‐33 and its implications in tumor immunity. Front Immunol. 2018;9:2601. 30483263 10.3389/fimmu.2018.02601 PMC6242976

[136] Cayrol C , Girard J‐P . IL‐33: an alarmin cytokine with crucial roles in innate immunity, inflammation and allergy. Curr Opin Immunol. 2014;31:31–37. 25278425 10.1016/j.coi.2014.09.004

[137] Lu B , Yang M , Wang Q . Interleukin‐33 in tumorigenesis, tumor immune evasion, and cancer immunotherapy. J Mol Med (Berl). 2016;94(5):535–543. 26922618 10.1007/s00109-016-1397-0

[138] Wasmer MH , Krebs P . The role of IL‐33‐dependent inflammation in the tumor microenvironment. Front Immunol. 2017;7:682. 28119694 10.3389/fimmu.2016.00682 PMC5220330

[139] Larsen KM , Minaya MK , Vaish V , Peῆa MMO . The role of IL‐33/ST2 pathway in tumorigenesis. Int J Mol Sci. 2018;19(9):2676. 10.3390/ijms19092676 PMC6164146 30205617

[140] Andreone S , Gambardella AR , Mancini J , et al. Anti‐tumorigenic activities of IL‐33: a mechanistic insight. Front Immunol. 2020;11:571593. 33329534 10.3389/fimmu.2020.571593 PMC7734277

[141] Cayrol C , Girard J‐P . IL‐33: an alarmin cytokine with crucial roles in innate immunity, inflammation and allergy. Curr Opin Immunol. 2014;31:31–37. 25278425 10.1016/j.coi.2014.09.004

[142] Hui E . Immune checkpoint inhibitors. J Cell Biol. 2019;218:740–741. 30760493 10.1083/jcb.201810035 PMC6400575

[143] Neeper M , Schmidt AM , Brett J , et al. Cloning and expression of a cell surface receptor for advanced glycosylation end products of proteins. J Biol Chem. 1992;267:14998–15004. 1378843

[144] Hudson BI , Lippman ME . Targeting RAGE signalling in inflammatory disease. Annu Rev Med. 2018;69:349–364. 29106804 10.1146/annurev-med-041316-085215

[145] El‐Far AH , Sroga G , Jaouni SKA , Mousa SA . Role and mechanisms of RAGE‐ligand complexes and RAGE‐inhibitors in cancer progression. Int J Mol Sci. 2020;21(10):3613. 10.3390/ijms21103613 PMC7279268 32443845

[146] Taguchi A , Blood DC , del Toro G , et al. Blockage of RAGE‐amphoterin signalling suppresses tumour growth and metastases. Nature. 2000;405:354–360. 10830965 10.1038/35012626

[147] Logsdon CD , Fuentes MK , Huang EH , Arumugam T . RAGE and RAGE ligands in cancer. Curr Mol Med. 2007;7:777–1097. 18331236 10.2174/156652407783220697

[148] Perkins TN , Donnell ML , Oury TD . The axis of the receptor for advanced glycation endproducts in asthma and allergic airway disease. Allergy. 2021;76(5):1350–1366. 32976640 10.1111/all.14600

[149] Hsieh HL , Schafer BW , Sasaki N , Heizmann CW . Expression analysis of S100 proteins and RAGE in human tumors using tissue microarrays. Biochem Biophys Res Commun. 2003;307:375–381. 12859967 10.1016/s0006-291x(03)01190-2

[150] Kang R , Tang D , Schapiro NE , et al. The receptor for advanced glycation end products (RAGE) sustains autophagy and limits apoptosis, promoting pancreatic tumor cell survival. Cell Death Differ. 2010;17:666–676. 19834494 10.1038/cdd.2009.149 PMC3417122

[151] Chen CZ , Li L , Lodish HF , Bartel DP . MicroRNAs modulate hematopoietic lineage differentiation. Science. 2004;303:83–86. 14657504 10.1126/science.1091903

[152] Schickel R , Boyerinas B , Park SM , Peter ME . MicroRNAs: key players in the immune system, differentiation, tumorigenesis and cell death. Oncogene. 2008;27(45):5959–5974. 18836476 10.1038/onc.2008.274

[153] Haj‐Salem I , Fakhfakh R , Bérubé JC , et al. MicroRNA‐19a enhances proliferation of bronchial epithelial cells by targeting TGFbetaR2 gene in severe asthma. Allergy. 2015;70(2):212–219. 25443138 10.1111/all.12551

[154] Solberg OD , Ostrin EJ , Love MI , et al. Airway epithelial miRNA expression is altered in asthma. Am J Respir Crit Care Med. 2012;186(10):965–974. 22955319 10.1164/rccm.201201-0027OC PMC3530212

[155] Biton M , Levin A , Slyper M , et al. Epithelial microRNAs regulate gut mucosal immunity via epithelium‐T cell crosstalk. Nat Immunol. 2011;12(3):239–246. 21278735 10.1038/ni.1994

[156] Simpson LJ , Patel S , Bhakta NR , et al. A microRNA upregulated in asthma airway T cells promotes TH2 cytokine production. Nat Immunol. 2014;15(12):1162–1170. 25362490 10.1038/ni.3026 PMC4233009

[157] Karo‐Atar D , Itan M , Pasmanik‐Chor M , Munitz A . MicroRNA profiling reveals opposing expression patterns for miR‐511 in 624 alternatively and classically activated macrophages. J Asthma. 2015;52(6):545–553. 25405361 10.3109/02770903.2014.988222

[158] Lu TX , Lim EJ , Itskovich S , et al. Targeted ablation of miR‐21 decreases murine eosinophil progenitor cell growth. PLoS One. 2013;8(3):e59397. 23533623 10.1371/journal.pone.0059397 PMC3606295

[159] Collison A , Herbert C , Siegle JS , Mattes J , Foster PS , Kumar RK . Altered expression of microRNA in the airway wall in chronic asthma: miR‐ 126 as a potential therapeutic target. BMC Pulm Med. 2011;11:29. 21605405 10.1186/1471-2466-11-29 PMC3116478

[160] Mattes J , Collison A , Plank M , Phipps S , Foster PS . Antagonism of microRNA‐126 suppresses the effector function of TH2 cells and the development of allergic airways disease. Proc Natl Acad Sci U S A. 2009;106(44):676–18704. 10.1073/pnas.0905063106 PMC2773983 19843690

[161] Lu C , Zhou D , Wang Q , et al. Crosstalk of MicroRNAs and oxidative stress in the pathogenesis of cancer. Oxid Med Cell Longev. 2020;2020:2415324. 32411322 10.1155/2020/2415324 PMC7204110

[162] Johansson K , Weidner J , Rådinger M . MicroRNAs in type 2 immunity. Cancer Lett. 2018;425:116–124. 29604393 10.1016/j.canlet.2018.03.036

[163] Deng Z , Rong Y , Teng Y , et al. Exosomes miR‐126a released from MDSC induced by DOX treatment promotes lung metastasis. Oncogene. 2017;36(5):639–651. 27345402 10.1038/onc.2016.229 PMC5419051

[164] de Leeuw DC , Denkers F , Olthof MC , et al. Attenuation of microRNA‐126 expression that drives CD34+38‐ stem/progenitor cells in acute myeloid leukemia leads to tumor eradication. Cancer Res. 2014;74(7):2094–2105. 24477595 10.1158/0008-5472.CAN-13-1733

[165] Stittrich AB , Haftmann C , Sgouroudis E , et al. The microRNA miR‐182 is induced by IL‐2 and promotes clonal expansion of activated helper T lymphocytes. Nat Immunol. 2010;11(11):1057–1062. 20935646 10.1038/ni.1945

[166] Cho S , Wu CJ , Yasuda T , et al. miR‐23 approximately 27 approximately 24 clusters control effector T cell differentiation and function. J Exp Med. 2016;213(2):235–249. 26834155 10.1084/jem.20150990 PMC4749926

[167] Pua HH , Steiner DF , Patel S , et al. MicroRNAs 24 and 27 suppress allergic inflammation and target a network of regulators of T helper 2 cell‐associated cytokine production. Immunity. 2016;44(4):821–832. 26850657 10.1016/j.immuni.2016.01.003 PMC4838571

[168] Tomita M , Tanaka Y , Mori N . MicroRNA miR‐146a is induced by HTLV‐1 tax and increases the growth of HTLV‐1‐infected T‐cells. Int J Cancer. 2012;130(10):2300–2309. 20017139 10.1002/ijc.25115

[169] Mo MH , Chen L , Fu Y , Wang W , Fu SW . Cell‐free circulating miRNA biomarkers in cancer. J Cancer. 2012;3:432–448. 23074383 10.7150/jca.4919 PMC3471083

[170] Kawaguchi T , Komatsu S , Ichikawa D , et al. Clinical impact of circulating miR‐221 in plasma of patients with pancreatic cancer. Br J Cancer. 2013;108(2):361–369. 23329235 10.1038/bjc.2012.546 PMC3566805

[171] Nguyen HC , Xie W , Yang M , et al. Expression differences of circulating microRNAs in metastatic castration resistant prostate cancer and low‐risk, localized prostate cancer. Prostate. 2013;73(4):346–354. 22887127 10.1002/pros.22572 PMC3980954

[172] Taylor DD , Gercel‐Taylor C . MicroRNA signatures of tumor‐derived exosomes as diagnostic biomarkers of ovarian cancer. Gynecol Oncol. 2008;110(1):13–21. 18589210 10.1016/j.ygyno.2008.04.033

[173] Paladini L , Fabris L , Bottai G , Raschioni C , Calin GA , Santarpia L . Targeting microRNAs as key modulators of tumor immune response. J Exp Clin Cancer Res. 2016;35:103. 27349385 10.1186/s13046-016-0375-2 PMC4924278

[174] Trang P , Wiggins JF , Daige CL , et al. Systemic delivery of tumor suppressor microRNA mimics using a neutral lipid emulsion inhibits lung tumors in mice. Mol Ther. 2011;19(6):1116–1122. 21427705 10.1038/mt.2011.48 PMC3129804

[175] Probst HC , McCoy K , Okazaki T , Honjo T , van den Broek M . Resting dendritic cells induce peripheral CD8+ T cell tolerance through PD‐1 and CTL‐4. Nat Immunol. 2005;6:280–286. 15685176 10.1038/ni1165

[176] Chen X , Shao Q , Hao S , et al. CTLA‐4 positive breast cancer cells suppress dendritic cells maturation and function. Oncotarget. 2017;8:13703–13715. 28099147 10.18632/oncotarget.14626 PMC5355131

[177] Chen DS , Irving BA , Hodi FS . Molecular pathways: next‐generation immunotherapy – inhibiting programmed death‐ligand 1 and programmed death‐1. Clin Cancer Res. 2012;18(24):6580–6587. 23087408 10.1158/1078-0432.CCR-12-1362

[178] Hodi FS , O’Day SJ , McDermott DF , et al. Improved survival with ipilimumab in patients with metastatic melanoma. N Engl J Med. 2010;363:711–723. 20525992 10.1056/NEJMoa1003466 PMC3549297

[179] Rizvi NA , Mazières J , Planchard D , et al. Activity and safety of nivolumab, an anti‐PD‐1 immune checkpoint inhibitor, for patients with advanced, refractory squamous non‐small‐cell lung cancer (CheckMate 063): a phase 2, single‐arm trial. Lancet Oncol. 2015;16(3):257–265. 25704439 10.1016/S1470-2045(15)70054-9 PMC5726228

